# Chronic Paraquat Neurotoxicity and Dopaminergic Degeneration: A Mechanistic Review

**DOI:** 10.1002/cbdv.71676

**Published:** 2026-09-10

**Authors:** Saima Riaz, Shaukat Ali, Muhammad Summer, Amina Iqbal, Rimsha Ashraf, Ramzana Bibi

**Affiliations:** ^1^ Medical Toxicology and Biochemistry Laboratory Department of Zoology Government College University Lahore Pakistan

**Keywords:** dopamine catabolism, neurotoxins, oxidative stress, paraquat, Parkinson's disease

## Abstract

Parkinson's disease (PD) is a significant neurodegenerative disorder that affects 1%–2% of the entire global population. Despite its higher prevalence, an effective treatment that prevents or reverses neuronal damage is still not available. Clinical characteristics of PD include bradykinesia, postural instability, tremor, and rigidity. This disease is pathologically defined by progressive degeneration of dopaminergic neurons in the substantia nigra pars compacta (SNpc) and by Lewy pathology with aggregated α‐synuclein (oligomers), although the exact role of Lewy bodies (LB) in cell death is controversial. Like other neurodegenerative disorders, the etiology of PD is associated with genetic, environmental, and other unknown factors. Certain neurotoxins such as rotenone, maneb, 6‐OHDA, MPTP and paraquat are main factors in the causation of PD. Among them, paraquat (PQ) is strongly linked with PD because of its common use in developing countries. Many *in vitro* and *in vivo* studies have reported the occurrence of PD due to exposure to PQ. The apoptotic cell death caused by PQ is associated with oxidative stress, ER stress, catabolic changes in dopamine, mitochondrial dysfunction, and so forth. The current review will summarize the available scientific evidence concerning the use and impacts of chronic exposure to PQ in the light of PD.

## Graphical abstract

Occurrence of Parkinson's disease (PD) based on several neurotoxins. Advanced and comprehensive mechanisms based on Paraquat‐induced PD. Mechanism of oxidative stress, ROS and RNS formation, and death of dopaminergic neurons. The modulation of genes and proteins by paraquat leads to Parkinsonism.

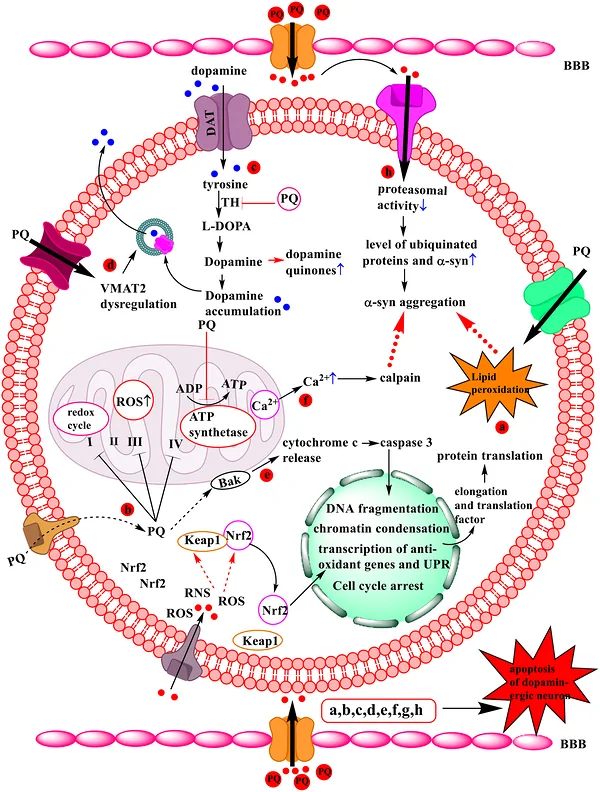

**Abbreviations**
6‐OHDA: 6‐hydroxydopamine
ALDH: Aldehyde dehydrogenase
ARE: Antioxidant response element
BAK: BCL‐2 antagonist/killer pathway
BAX: BCL‐2 associated X protein
BCL‐2: B‐cell lymphoma 2
COMT: Catechol‐O‐methyltransferase
CS_2_
: Carbon disulfide
DA: Dopamine
DAT: Dopamine transporter
DJ‐1: Daisuke‐Junko‐1 gene
DOPAC: 3‐4, dihydroxyphenyl acetic acid
ER stress: Endoplasmic reticulum stress
GSH: Glutathione
HO‐1: Heme oxygenase‐1
IL‐1β: Interleukin‐1 beta
IL‐6: Interleukin‐6
JNK: c‐Jun‐N‐terminal kinase pathway
Keap1: Kelch‐like ECH‐associated protein 1
L‐dopa: Levodopa or l‐3,4‐dihydroxyphenylalanine
LXR: Liver X receptor pathway
MAO: Monoamine oxidase
MMP: Matrix metalloproteinases
MOMP: Mitochondrial outer membrane permeabilization
NF‐kβ: Nuclear factor kappa B
NM: Neuromelanin
NO: Nitric oxide
Nrf2: Nuclear factor erythroid 2‐related factor 2
PINK1: PTEN‐induced putative kinase 1
PPE: Personal protective equipment
RNS: Reactive nitrogen species
ROS: Reactive oxygen species
Smac: Second‐mitochondria‐derived activator of caspases
SNCA: Synuclein alpha
SNpc: Substantia nigra pars compacta
TNF‐α: Tumor necrosis factor alpha
VMAT2: Vesicular monoamine transporter 2

## 1 Introduction

Parkinson's disease (PD) is the second most common neurodegenerative disorder [1] after Alzheimer's disease, having progressive and severe loss of dopaminergic neurons in the substantia nigra pars compacta (SNpc) [2]. The clinical manifestations, which include bradykinesia [3], hypokinesia, resting tremors, and rigidity, occur once the loss of these cells exceeds 70% [4]. This condition was first described by James Parkinson in “An Essay on the Shaking Palsy” in 1817 [5]. It is a significant medical and public health issue and poses emotional, physical, and financial burdens not only on patients, relatives, and family members but also on society [6]. It is a multi‐system and complicated disorder having both motor and non‐motor symptoms; the slow‐developing non‐motor complications include sleep behavior disorder, autonomic disorders, rapid eye movement, and gastrointestinal problems [7, 8].

The occurrence of PD differs across different geographic regions [9], but globally the estimated number of people with PD is 6.1 million [10], with 1.02 million cases reported each year [11, 12]. Almost 4.4 million cases of PD were reported globally in 2005, and this figure is projected to double to almost 9 million in the year 2030. The incidence of this disease is almost 0.3% in industrialized countries [13]. Furthermore, the Global Burden of Disease study has found that PD has been experiencing an increase in the prevalence rate among older people [14]. People below the age of 40 are less affected, and people above the age of 80 are most affected [9]. Furthermore, data have forecasted an increase in the cases of PD in North America to 1,238,000 by 2030, compared to 680,000 in the year 2010 [15]. In the United States, the prevalence of PD among individuals at the age of 65 or above is approximately 160 per 100,000 individuals, and approximately 60,000 new cases and above 20,000 deaths are reported each year. Meanwhile, the Parkinson's Disease Foundation 2022 reported that almost 1 million people are living with PD in America, and it costs almost 52 billion dollars annually, directly or indirectly [16].

There are many factors, such as genetic mutations, metabolic defects, and exposure to environmental agent sare linked with neurodegenerative disorders [17]. Potential determinants that lead to an increase in the burden of PD are the increasing population size of older age groups, unhealthy activities such as physical inactivity, some dietary practices, and exposure to industrial toxins. It is still an ongoing quest to identify nongenetic risk factors of PD, and a list of potential suspects includes microorganisms, air pollution, heavy metals, traumatic brain injury, alcohol consumption, electromagnetic fields, medications, and exposure to various pesticides, which attract the research community [18]. While rare monogenic forms of PD are particularly linked with mutations in several genes such as PINK1, DJ‐1, PARKIN, and alpha‐synuclein [19].

The possible correlation between long‐term organophosphate exposure and changes in the central cholinergic and dopaminergic systems, as well as the particular danger of agricultural workers having heightened risks of getting PD, was first elaborated in 1978 [20]. The US Research Council indicates that the number of chemicals, pesticides, or insecticides that can cause developmental disorders is up to 127 [21]. A single exposure to 1‐methyl‐4‐ phenyl‐1,2,3,6‐tetrahydropyridine (MPTP) has been shown to cause irreversible parkinsonism in human beings, rodents, and nonhuman primates by acting primarily on mitochondrial complex I. Some other well‐known chemicals that can cause PD include CS_2_, chronic exposure to manganese, dieldrin, O6HDA, rotenone, and most specifically, paraquat (PQ) [22].

Among them, PQ, which is a widely used herbicide [23, 24], has been under investigation in epidemiological and toxicological studies during the last 30 years [25]. The correlation between exposure to PQ and the development of PD has gained attention since this hypothesis was proposed in the mid‐1980s [26]. However, PQ has been a subject of research due to structural similarities with MPTP, which is used to induce PD in animal models [27]. PQ passes the Blood–Brain Barrier (BBB) via the same transporter used by neutral amino acids such as L‐valine and L‐dopa [28]. Within neurons, PQ causes oxidative stress by the generation of superoxide anions via redox cycling [29]. The high ROS production is specifically sensitive in dopaminergic neurons due to the metabolism of dopamine, which produces radicals of superoxide and hydrogen peroxide, which subsequently react with nitric oxides to produce very toxic peroxynitrite [30, 31]. The excessive formation of ROS overloads metabolic resources because the cell reacts to misfolded proteins, replaces lipids that are oxidized, and fixes DNA damage. Cells that are unable to cope with this oxidative stress default to apoptosis or necrosis in extreme conditions [3].

The Agricultural Health Study (USA) demonstrates that the risk of PD increases in direct proportion to the number of days of exposure to this pesticide [32]. Despite the prohibition of the use of PQ in Europe and some other places, it is still extensively used as an herbicide in third‐world countries [33, 34]. In these nations, the use of personal protective equipment is often ineffective, and any equipment can be dysfunctional. Moreover, such areas have a lack of awareness regarding personal protection and how to use hazardous chemicals [35]. In this review, we will examine the cellular and molecular alterations caused by PQ and the proper mechanism by which PQ causes PD. The emerging data from epidemiological, clinical, and experimental studies on the relationship between the most commonly employed herbicide, PQ, and PD pathology are discussed.

## 2 Pathology of PD

Complete development of PD constitutes motor symptoms such as bradykinesia, resting tremors, and so forth [36]. The nigral cells present in normal brains decline by a factor of 4.7%–6% during each decade between the fifth and the ninth decade of life, even though this reduction is not enough in order to bring about PD. The shared characteristics of PD are the loss of the neural pathway that connects substania nigra (SN) and the striatum, which are important brain areas in order to properly regulate normal motor movements [37]. Neurons of SNpc provide the striatum with its dopaminergic innervation through the nigrostriatal pathway [38]. The striatal dopamine deficiency is severe due to the gradual degeneration of this nigrostriatal dopaminergic pathway [11].

By the time the clinical symptoms of PD have completely appeared, almost 80% of the dopaminergic neurons in the SN have already been destroyed, hence leading to the impairment of production and secretion of DA from the striatal nerve endings [39]. In addition to SN neuronal loss, other notable pathological characteristics of PD are the formation of Lewy bodies (LB) [40, 41], which are cytoplasmic inclusions that form in surviving dopaminergic neurons. These cytoplasmic inclusions are present in different regions of the brain such as magnocellular basal forebrain nuclei and cortex, along with dopaminergic neurons of the SN in PD [42]. However, many different clinical syndromes are also linked with intracellular inclusions of α‐synuclein (synucleinopathies) [39].

## 3 Etiology of PD

There is a significant amount of evidence showing the multifactorial etiology of PD, which involves both genetic mutations and environmental factors [17, 20]. The role of genetics in the development of PD has been analyzed in twin studies: case control and in investigating mutations in α‐synuclein, PINK1, Parkin, DJ‐1, and dardarin [43]. Moreover, all cases of PD cannot be explained entirely by means of inheritance. Actually, all LBs contain α‐synuclein, even in most cases of idiopathic PD without mutations in α‐synuclein, hence suggesting that other processes can trigger changes in conformation and the resultant protein aggregation [44, 45]. Many environmental factors have also been linked to cause PD either in the regulation of disease onset or the progression of disease. The environmental factors are reported to induce nigrostriatal injury and PD include solvents, carbon monoxide, pesticides, metals [46]. Further, epidemiological research results indicate a correlation between elevated risks of PD and certain environmental factors such as residence in rural areas, farming [47], and consuming water from wells, as well as exposure to agricultural chemical compounds including PQ. Taking into consideration the complications to public health due to risk factors of PD, the research on the nature of environmental factors that are involved in the etiology of this disease has become an emerging concern of both the medical and scientific community [48].

## 4 Neurotoxins and Pesticides Involved in PD

**FIGURE 1:**
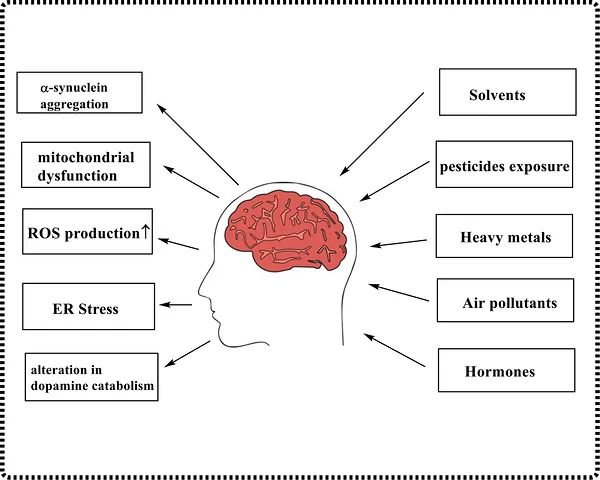
Neurotoxins and their effects in neurons.

Several pesticides and neurotoxins can cause PD [4], and they have been discussed briefly in the etiology of PD and are shown in Figure 1. These factors behave differently in the occurrence of familial, idiopathic, and secondary or toxin‐induced Parkinsonism. Genetic mutations are important in the occurrence of familial disease. The most commonly occurring form is idiopathic PD with unknown causes [49, 50]. Although mutations in genes have less role in the occurrence of disease, these genetic models are very important in understanding the rare monogenic forms of disease. Whereas secondary or toxin‐induced PD is associated with exposure to pesticides, metals, drugs, trauma, or other toxicants [50]. The epidemiological research indicates that the farming community has a higher prevalence of toxin‐induced PD [51]. Additionally, exposure to pesticides or herbicides leads to a three‐ to four fold increase in the occurrence of developing PD; this enhanced risk endorses the role of environmental agents in the pathogenesis of PD [52]. Approved drugs, pesticides, industrial chemical compounds, hormones, and heavy metals modulate gene expression with a wide range of regulatory mechanisms, and influence the dopaminergic system; hence these agents might be related to various pathological conditions such as PD [53]. Many PD‐like experimental models that are triggered by various neurotoxins such as insecticides or pesticides and resemble the molecular, pathological, and behavioral features of disease are shown in Table 1.

**TABLE 1:** Neurotoxins and their effects in neurons in *in vivo* experimental models.

Neurotoxins	Route of induction	Performance	Degradation of DA neuron	α‐syn/LB	Experimental model	Cellular and molecular events	Model features	Limitations	References
Rotenone	Intraperitoneal, subcutaneous, intravenous, and oral administration	Slow gait, limb tremors, and stiff movements	Degradation and death of neurons	+/+	Zebrafish, rodents, and drosophila	Retards mitochondrial complex I, energizes Microglia, proteasomal function impairment, ROS↑, and autophagy	Best for chronic PD modeling, helpful in detecting cases of the peripheral nervous system in early PD stages	Model animals fail to mimic the chronic form of human PD due to its rapid decay, mild appearance of symptoms, and rapid healing	[54, 55, 56]
6‐OHDA	Intracerebral and stereotactic injections	Movement disorders on the opposite side of injection	Degradation and demise of neurons	+/−	Rats, mice, cats, dogs, and monkeys	Inhibits mitochondrial complex I, ROS↑, and antioxidant enzymes↓	Suitable for modeling human PD motor symptoms, and helpful to investigate the effect of drug treatment	Dyskinesia expresses as lateral rotation, and it does not mimic the fairly complex and variable human PD behavior. No production of LBs, acute model that leads to shorter and severe onset of disease	[57, 58]
MPTP	Subcutaneous, intramuscular, intravenous, and intraperitoneal	Slow movement, reduced activity	Degeneration and loss of DA neurons	+/−	Non‐human primates, Mice	Inhibits mitochondrial complex I, ATP↓, ROS↑	Suitable for modeling different stages of PD, helpful for investigating mitochondrial dysfunction	Production of LBs is controversial. The reagent is toxic and does not mimic symptoms of human PD, especially motor symptoms	[59, 60]
PQ	Intraperitoneal, subcutaneous, intracerebral, and oral–nasal administration	Reduction in activity and shaking of limbs	Demise of neurons	+/+	Mice and rats	Dysfunction of mitochondria, interferes with glutathione's redox reactions, proteasomal activity↓, and antioxidant activity↓	α‐syn aggregation is observed. LB production is suitable for modeling the chronic form of PD, and PD can be assessed at preclinical stages	Extremely toxic to people, dosing procedure is very specified	[61, 62, 63, 64]
Maneb	Intraperitoneal and subcutaneous	Gradual decline in movement upon administration with PQ	Degeneration of neurons	—	Mice and rats	Inhibition of mitochondrial complex III, ROS↑, and GSH antioxidant defenses↓	Appropriate to observe pathological characteristics of glial activation, slight motor impairment, and oxidative stress	Does not completely recapitulate PD, leads to chronic PD when administered with other toxins, particularly PQ	[65, 66, 67]
Dieldrin	Intraperitoneal and oral administration	Slow movement	Degeneration and demise of neurons	+/+	Mice and rats	Reduction in oxidative phosphorylation	Inhibits proteasomal degradation, interferes with metabolism of dopamine and vascular storage	Fails to mimic human PD like model completely	[64, 68]

## 5 Paraquat

PQ, belonging to the wide‐spectrum bipyridylium group of herbicides [69], is nonselective, acts rapidly, and has the ability to interfere with intracellular transportation in plants, disturb plant organelles, and eventually cause cell death [30]. It is used on a large scale to control broad‐leaved weeds [70] and grasses in various agricultural and non‐agricultural settings such as cotton, maize, sugarcane, and soybeans [20, 71]. It is also a dangerous herbicide that induces acute poisoning and even death. Ingestion, exposure to the skin, and inhalation can be some of the most common pathways of PQ exposure that may lead to poisoning either intentionally or through accidental contact [24]. The herbicide PQ has been the primary subject of experimental studies to examine its effects on the lungs, liver, muscles, kidneys, and cardiovascular system because of its systemic toxicity and capacity to cause fatalities after acute exposure [71, 72].

**FIGURE 2:**
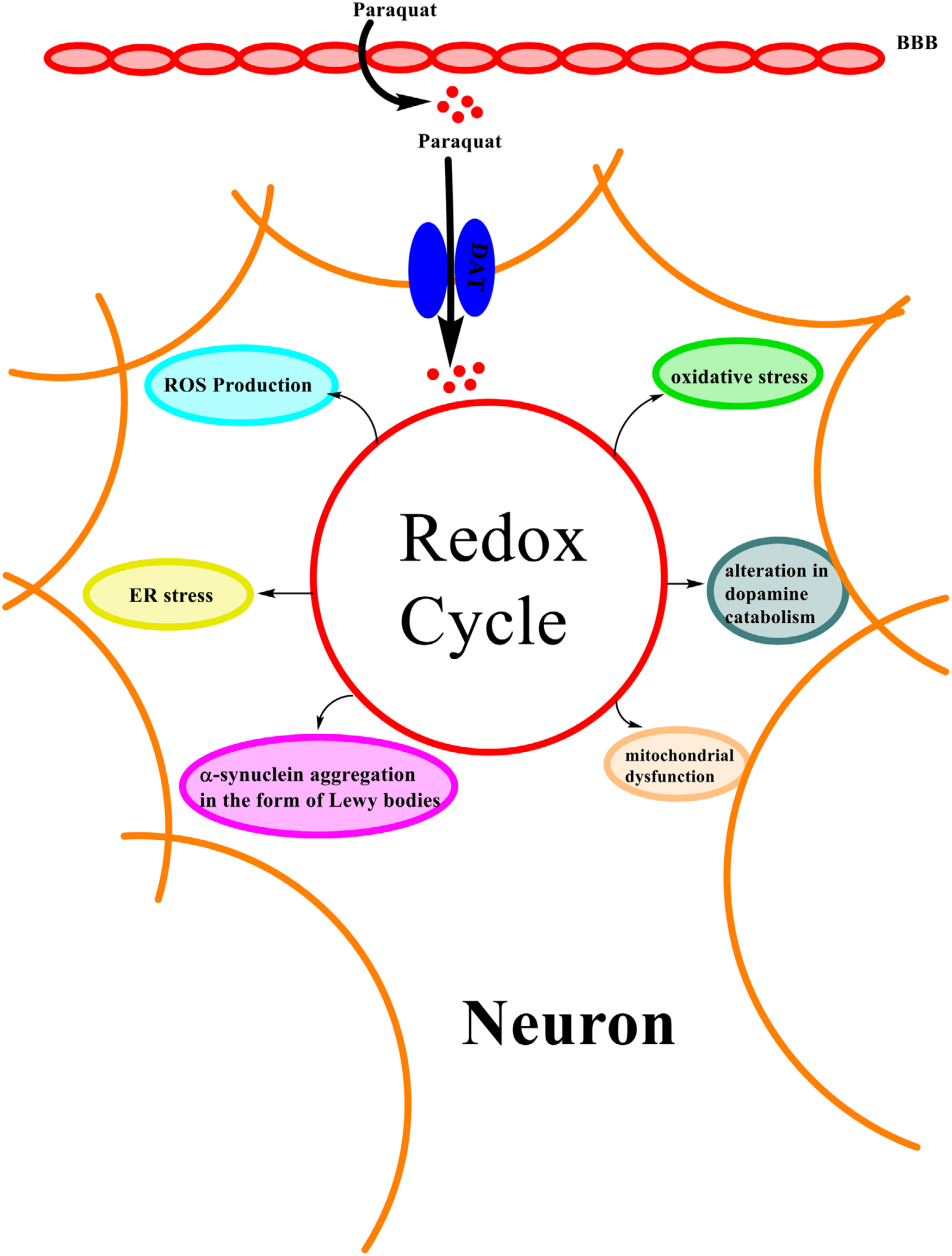
Cellular and molecular processes involved in PQ‐induced damage. After passing the BBB, PQ enters the neuron via DAT and triggers a cycle that induces oxidative stress, mitochondrial dysfunction, and protein aggregation.

PQ has been considered a potential model of PD because it shares some biochemical and molecular similarities with 1‐methyl‐4‐phenylpyridinium (MPP+), which is an active metabolite of MPTP that produces Parkinson‐related symptoms in both animal and human PD models [73]. Over the years, research studies have indicated that exposure to PQ raises the risk of PD [3]. Within neurons, PQ induces oxidative stress by ROS production, lipid peroxidation, α‐synucelin aggregation, ER stress, and modulation of dopamine catabolism [74], as shown in Figure 2. These alterations result in the death of dopaminergic neurons [75]. In addition to cellular and molecular changes associated with PQ, this review provides complete information about exposure to PQ and the risk of PD. We will also discuss how changes induced by PQ may possibly trigger apoptotic processes leading to cell death.

## 6 Mechanism of PQ Toxicity to Induce PD

### 6.1 PQ and BBB

PQ has the ability to penetrate the BBB. The penetration is indeed inefficient and slow, but a measurable amount of this herbicide has been found in the CNS following systemic injection [69]. Research studies have confirmed that PQ passes through the BBB of Wistar‐derived Alderley Park male rats when they were treated with a subcutaneous dose of PQ that was ^14^C labeled [20]. Following the administration, the concentration of PQ in the brain was at its peak in the 1st h of administration, and after that it abruptly decreased, leaving behind only small traces of pesticide [28]. The researchers proposed a possible association between the health condition of microvascular endothelial cells of the brain that constitute the BBB and minimal penetration of PQ [71]. Notably, PQ and endothelial cell interaction did not lead to the deterioration of BBB functioning by either PQ or any of its radicals. Upon entry into the CNS, PQ tends to be linked with five important structures, namely the anterior section of the olfactory bulb, pineal gland, area postrema, cerebral ventricles, and hypothalamus [76]. Notably, this distribution is associated proximally with the cerebral capillary network. In addition to this, PQ also exists in the ventral midbrain, and it appears to have a half‐life of around 4 weeks [77].

It has been reported that PQ is taken up into dopaminergic neurons by the dopamine transporter (DAT) because of the structural resemblance between PQ and MPP^+^, which can also access the striatal cells in an Na^+^‐dependent manner [62]. But Richardson and his colleagues dismissed the involvement of DAT in the process of dopamine penetration. They explained that PQ is not a substrate of DAT due to its inability to block the uptake of dopamine mediated by DAT and induce dopaminergic destruction [78]. The clarification and confirmation of the molecular pathways utilized by PQ to access other brain regions, including dopaminergic areas, are still outstanding queries; hence, additional research is needed to know how PD‐like changes develop [79].

### 6.2 PQ Induces α‐Synuclein Pathology

**FIGURE 3:**
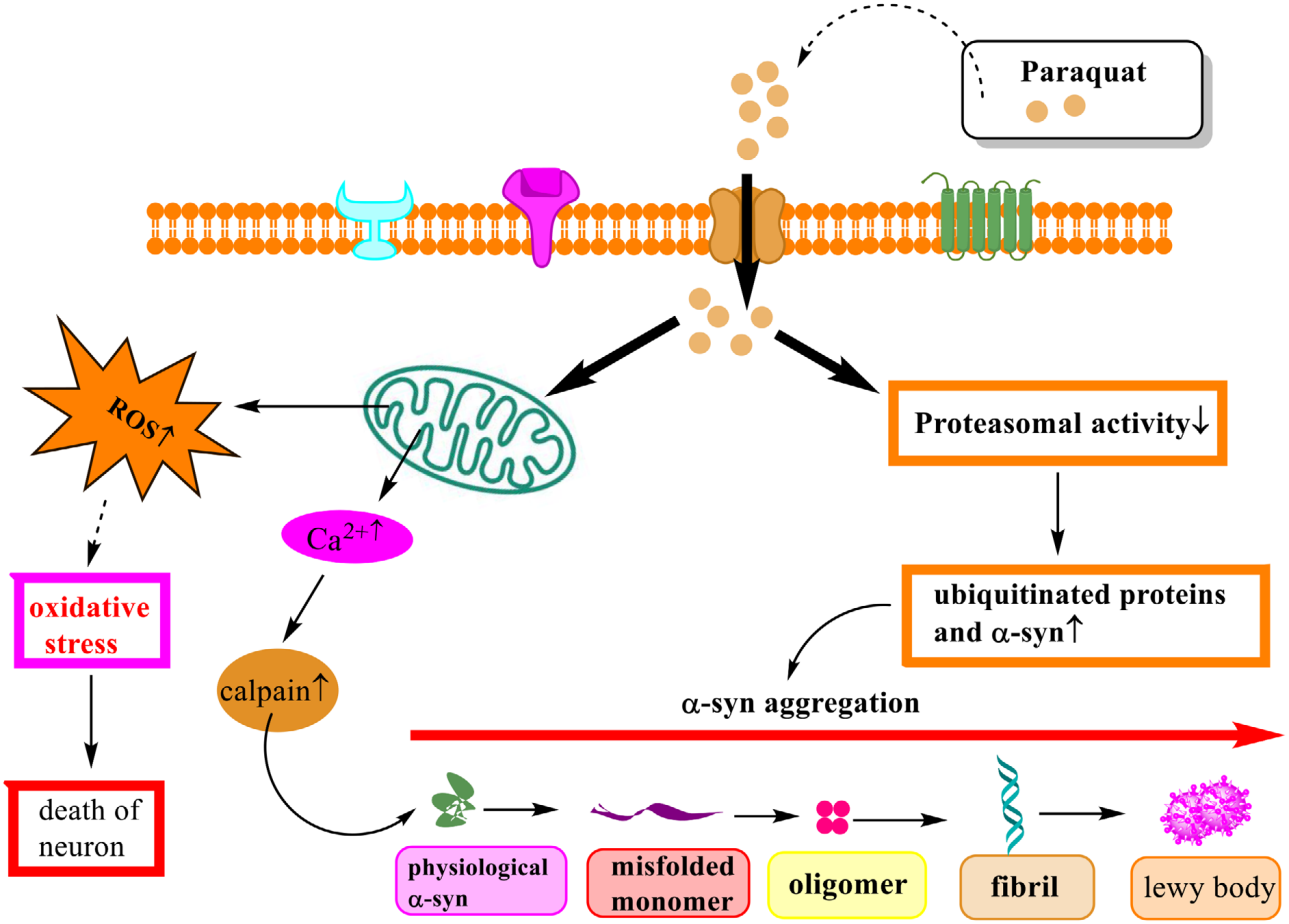
Accumulation and aggregation of α‐synuclein in neurons.

In most of the cases of PD patients, neuronal inclusions termed LBs are found in numerous dopaminergic SNpc cells [80, 81]. An LB is composed primarily of an aggregation of an intra‐cytoplasmic protein termed α‐synuclein [41, 82]. This α‐synuclein is a highly dynamic and intrinsically disordered protein that can be able to adopt either a soluble monomeric form or a helical multimeric form [83]. Nevertheless, α‐synuclein can be capable of transforming from a monomeric conformation into various oligomeric and aggregated forms [84, 85], such as fibrils and spherical aggregates that are constructed via enlisting α‐synuclein monomeric subunits [39, 86], as shown in Figure 3. The precise role of α‐synuclein is still undetermined. Because this protein is primarily found in the presynaptic terminal of neurons, it might be involved in plasticity of synapse and release of neurotransmitters [87, 88].

At cellular levels, this protein is predominantly expressed in the Golgi apparatus, presynaptic terminals of neurons, endoplasmic reticulum, mitochondria, as well as the endo‐lysosomal system [41, 89]. It is not yet understood what the specific physiological role of α‐synuclein is in any of the subcellular compartments. But α‐synuclein has the ability to bind well with synphilin‐1, which is an adapter protein and tethers α‐synuclein to the proteins of the cytoplasm that mediate transport of vesicles and cytoskeleton activity. According to the cumulative research, it has been suggested that the accumulation of α‐synuclein (oligomers) influences neuronal function [90, 91] and may cause neurotoxicity. Furthermore, α‐synuclein binds to complex I of the electron transport chain (ETC) and leads to increased formation of ROS, which further changes the expression of genes encoded in the mitochondrial genome and causes fragmentation of mitochondria [89].

Recently, it has been strongly indicated that there is an interaction of α‐synuclein with environmental toxicants like PQ, leading to an elevated α‐synuclein propensity to accumulate and to oligomerize [92]. In vitro studies have reported conformational modifications in α‐synuclein by using PQ, and it enhances the rate of fibrillation of α‐synuclein in a dose‐dependent manner [93], as shown in Table 2. Even though the process of oligomerization of this protein has not been fully elucidated, it has been shown that the production of α‐synuclein radicals may be an essential mechanism. The herbicide, PQ produced the radicals of α‐synuclein in the midbrain of rats and mice via two important mechanisms: (i) the stimulation of NADPH oxidase and activation of nitric oxide synthase (iNOS) in the microglia to generate peroxynitrite (ONOO^−^) [94] and (ii) the release of cytochrome c out of the mitochondria into the cytosol to trigger peroxidase activity [95]. Apart from all this, additional research is required to determine the importance of α‐synuclein in neurodegeneration caused by PQ.

**TABLE 2:** Exposure of different doses of paraquat in different in vitro and in vivo experimental models and status of α‐synuclein aggregation.

Model (organisms/cells)	PQ dose	α‐synuclein status	Findings	References
SH‐SY5Y cells	Not specified	Increased rate of intracellular α‐synuclein concentration	Only monomers identified; no oligomers or fibrils were detected	[96, 97]
Mice (wild‐type)	Intraperitoneal, 10 mg/kg once a week for 3 weeks	∼1.5‐fold increase in α‐synuclein in ventral mesencephalon and frontal cortex after 48 h of treatment	Back to baseline level within a week after treatment	[93, 98]
Mice (wild‐type)	Intraperitoneal, 10 mg/kg, twice a week for 4 weeks	∼2.1‐fold increase in α‐synuclein in the striatum	Regional variations in α‐synuclein expression were detected	[99]
Mice (overexpressing human α‐syn)	Intraperitoneal, 10 mg/kg, once a week for 3 weeks	∼1.9‐fold increase in α‐synuclein accumulation in SNpc	Aggregation detected only in α‐syn overexpressing mice, and not in WT (wild‐type)	[73, 100, 101]
Mice (wild‐type)	Intraperitoneal, 10 mg/kg, once a week for 3 weeks	No aggregation of α‐synuclein observed	Proteinase K pretreatment used to visualize insoluble aggregates	[100, 102]

### 6.3 Oxidative Stress Induced by PQ

#### 6.3.1 Increased Lipid Peroxidation

Lipid peroxidation is a process that can be caused by oxidative stress [103] and destroys the cell membranes, lipoproteins, and other lipid‐containing products [104]. The brain is more vulnerable to this peroxidation of lipids because of the abundance of unsaturated fatty acids in the brain [3]. A meta‐analysis has found higher levels of malondialdehyde (MDA) [23], a product of lipid peroxidation, in the blood of patients with PD [105]. Other end products of the lipid peroxidation process, including Nε‐(carboxymethyl) lysine and 4‐hydroxynonenal (HNE), have also been detected in LB of brain tissues having PD. Furthermore, it was demonstrated that MDA levels were observed to be doubled in PQ (14 µM, 24 h) treated SK‐N‐SH cells [106]. The PQ‐treated brain of Drosophila also had increased levels of MDA [107, 108]. As MDA is a popular indicator of lipid peroxidation, the above findings indicate that the potential of PQ to cause oxidative stress results in higher lipid peroxidation in the brain [109, 110].

#### 6.3.2 Intracellular Increased Level of ROS

**FIGURE 4:**
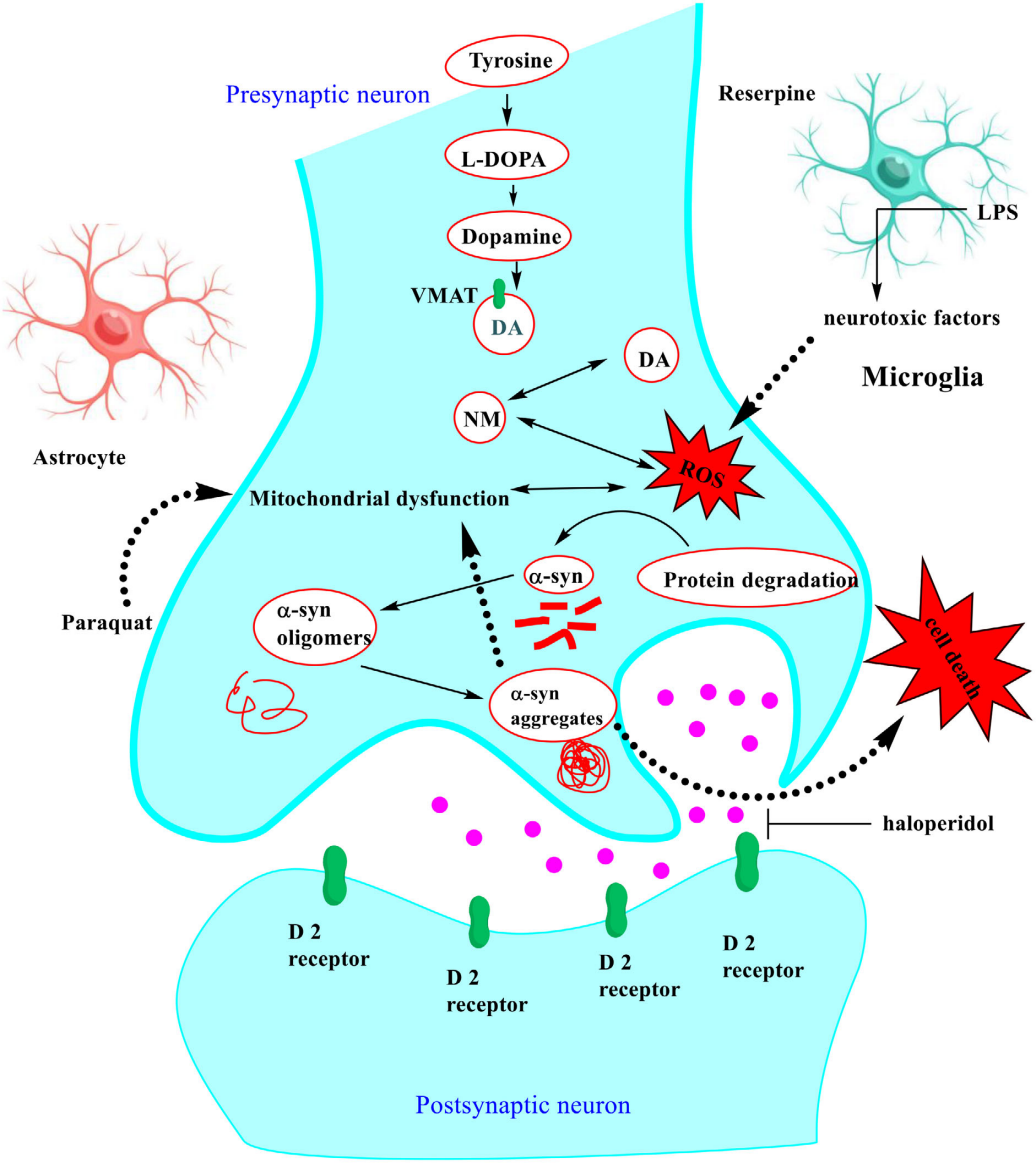
Summary of mechanisms that are known to produce reactive oxygen species and death of neurons in models of PD by PQ.

Almost all neurodegenerative disorders are characterized by oxidative stress [111]. Mitochondrial production of ROS and increased metabolism of molecular oxygen by the ETC have the potential to make various types of cells, especially neurons, extremely susceptible to oxidative stress [112]. Furthermore, the capacity of ROS to influence both the functioning of mitochondria and nucleic acids, thus altering the expression of genes and other cellular processes, will inevitably lead to severe cellular changes and cell death [113]. In PD, particularly, oxidative stress is associated with LB development because of SNCA aggregation [84] and dysfunction of the ubiquitin‐proteasome system (UPS) [20], as shown in Figure 3. Neurotoxins, especially PQ, result in the damage of dopaminergic neurons by triggering the mechanisms of oxidative stress due to an elevated amount of ROS, free radicals, and anions as shown in Figure 4 [102, 114].

#### 6.3.3 PQ and Mitochondrial Dysfunction

**FIGURE 5:**
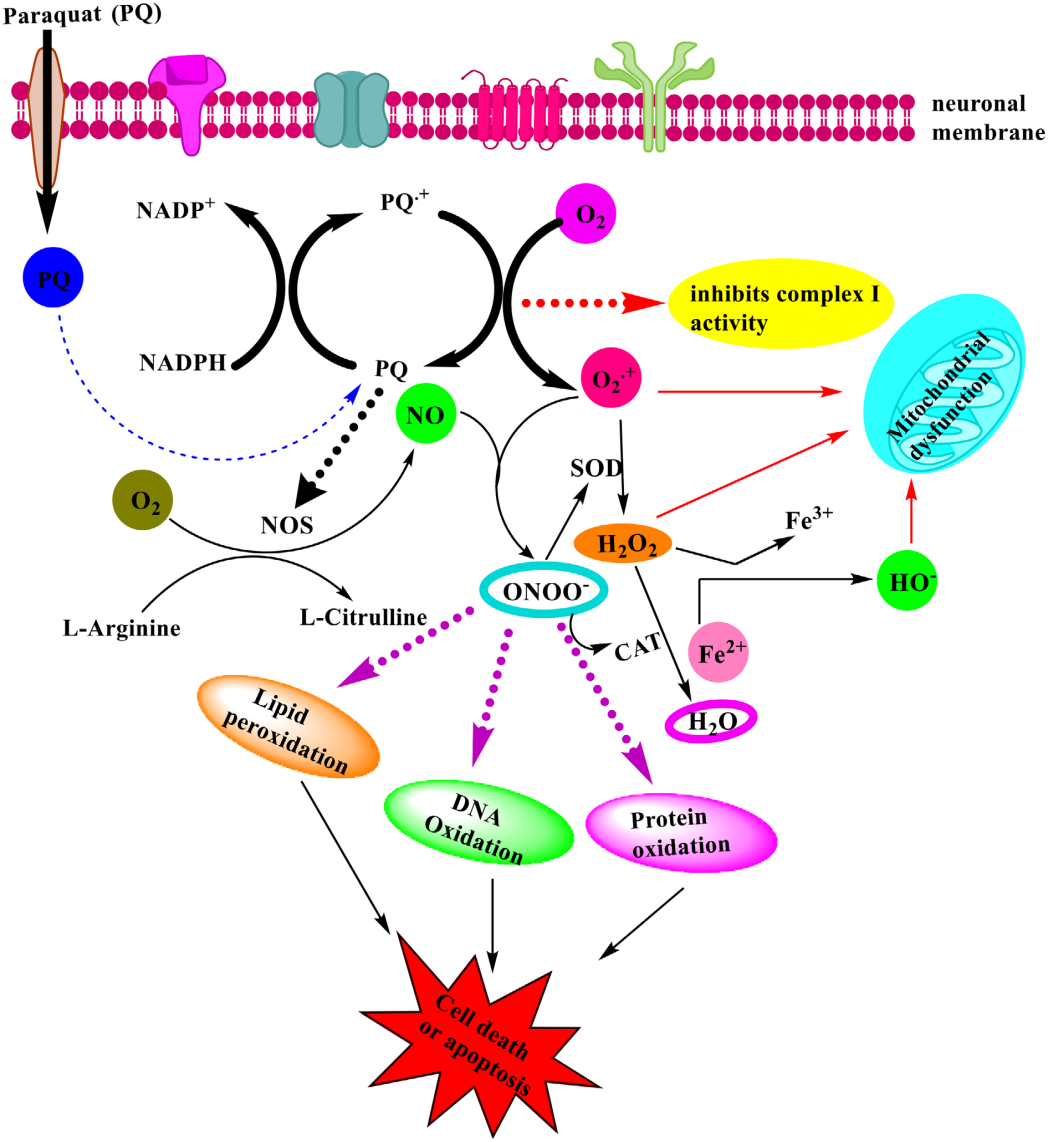
Representation of oxidative stress and mitochondrial dysfunction induced by PQ. PQ inhibits the activity of complex I by being converted into PQ^+^ via NAD(P)H reductase (cytochrome P450). In the presence of O_2_, PQ is oxidized with the production of O_2_^.^ This causes mitochondrial dysfunction when it is metabolized into H_2_O_2_ and OH^−,^ and this process results in the apoptosis of dopaminergic neurons. Furthermore, PQ also stimulates the production of NO by triggering the activation of NO synthase. This NO then reacts with O_2_^−.^ to produce ONOO^−^, which results in cell apoptosis.

Paraquat causes mitochondrial dysfunction by inhibiting the function of mitochondrial complex I in the SNpc of PD patients [115]. PQ cytotoxicity has a direct impact on this complex, and causes mitochondrial cytopathy. Mitochondrial complex I basically oxidizes NAD(P)H into NAD(P)^+^ by delivering electrons to ubiquinone in the ETC [53]. PQ^2+^ interferes with the oxidation of this NAD(P)H because it accepts electrons and becomes PQ^+^ via NAD(P)H‐cytochrome P450 reductase, which ultimately interferes with the role of complex I [7]. PQ^+^ produces superoxide radicals (O_2_^−.^) [116] by reacting with oxygen and accepting electrons from the reductant [117], and then oxidizes back into PQ^2+^, and this oxidation–reduction cycle occurs repeatedly by the same molecule of PQ^2+^ [118]. The reactive O_2_^−.^ initiates a familiar cascade of reactions that produce other ROS, including H_2_O_2_ and hydroxyl radical (OH^−^) [119], as shown in Figure 5. The ROS produced by PQ competing for electrons in the cellular redox cycle, which alters the redox environment, induces mitochondrial dysfunction and results in subsequent death of dopaminergic neuronal cells in a sequential way. It is postulated that this process results in the development of PD [18].

Various recent reports have shown that there is no inhibition of complex I after exposure to PQ, indicating that complex I may not be a critical target. Moreover, PQ can cause neurotoxic effects without inhibiting complex I [58]. Nevertheless, the possibility of PQ causing death of dopaminergic neurons remains a basic phenomenon through inhibition of complex I [120]. It has been established that this inhibition of complex I leads to respiratory disorder in the brain [121]. Research studies have identified a considerable reduction in complex I activity in the brain of rats after 2 h of the administration of PQ [122]. PQ also causes generation of ROS in the complex III of the ETC, and this process has been highlighted using the Drosophila model [73]. Enzymes that play the most crucial role in bioactivation and metabolism of PQ are cytochrome P450, nitric oxide synthase (NOS), thioredoxin reductase, and NADPH oxidase (NOX) [76, 123]. These enzymes donate electrons from NAD(P)H to PQ, which results in oxidative stress along with mitochondrial dysfunction [80, 121], as shown in Figure 5.

#### 6.3.4 Nrf2‐Keap1‐ARE Signaling Pathway's Downregulation

**FIGURE 6:**
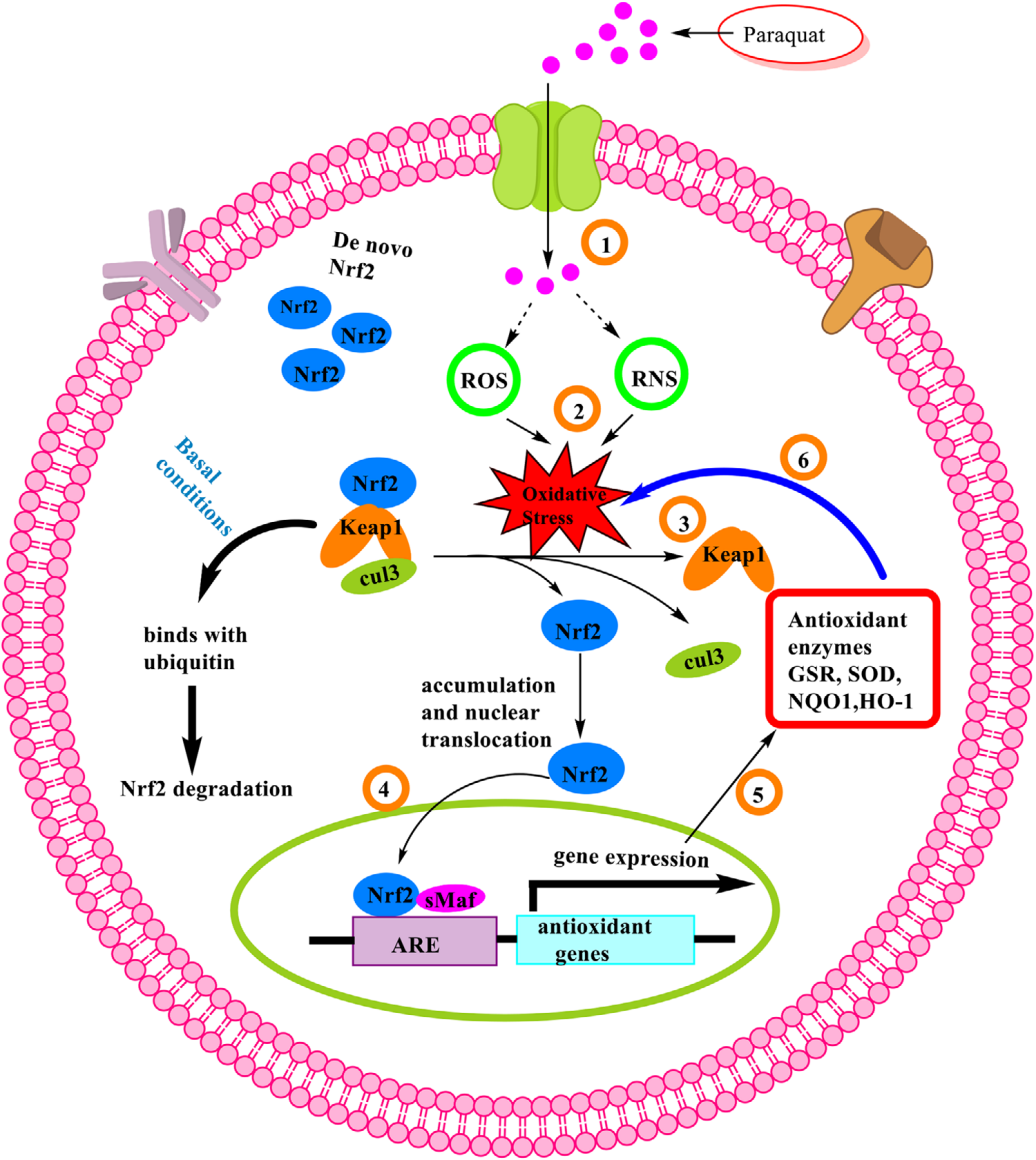
Oxidative stress produced by PQ leads to the transportation of Nrf2 into the nucleus, and this accumulation of Nrf2 is responsible for the production of antioxidant enzymes.

A major transcription factor, Nrf2, regulates multiple antioxidant defense pathways, which result in the generation of different types of antioxidant enzymes [122]. In the case of normal conditions, the intracellular supply of Nrf2 in the cytosol becomes reduced because it is rapidly degraded by the UPS [124], as shown in Figure 6. Nrf2 is degraded when it binds to Keap1, which is a cytoplasmic protein and resembles Kelch [7, 125]. Oxidative stress blocks Keap1‐mediated degradation of Nrf2, which results in active transport of Nrf2 into the nucleus, where it accumulates [126], as shown in Figure 6. Research studies’ results have shown that oxidative stress triggered by PQ could stimulate the Nrf2‐Keap1‐ARE pathway, leading to the development of downstream antioxidant response compounds such as HO‐1 mRNA and NQO1 [18, 127].

#### 6.3.5 Impaired Antioxidant Defense

Glutathione (GSH) is the systemic antioxidant present within the body [128] and is highly important in defending the cells against RNS and ROS [129]. But reduced levels of GSH in the SNpc of PD patients’ brains have been reported. Reduced GSH in the blood was also observed in the cases of PD [130]. This decrease in the amount of GSH has been observed to hamper the antioxidant defense mechanism of cells [131]. Research studies that relate exposure to PQ and reduction in antioxidant enzymes are illustrated in Table 3. The loss of this antioxidant in the brain of patients with PD could be attributed to a reduction in the production of GSH [132]. Due to this, it may be expected that there must be a decline in the functions of glutamate–cysteine ligase (GCL), which is a rate‐limiting enzyme in the production of GSH. It is true that the activity of GCL turned out to be diminished in many parts of the brain due to aging effects [133].

**TABLE 3:** Effects of exposure and duration of PQ on antioxidant enzymes.

Sr No.	Model organism	PQ dose and duration	Effect on antioxidants	References
1	Rats (postnatal+ adult exposure)	Intraperitoneal; 5 mg/kg, twice a week for 12 weeks	45% reduction of GSH in nigrostriatal tissues	[137]
2	GCL knockout mice	Not determined	60% decrease in GSH content in the striatum	[104, 138]
3	PC12 cells (differentiated)	150 µM for 24 h	30% decrease in GCL activity	[139, 140]
4	Adult Wistar rats	Intrastriatally, 2.5 µg/10 µL for 24 h	GSSG↑, GSSG/GSH ratio↑ in bilateral cortex	[125, 141]
5	Human (post‐mortem PD brain)	Not determined	Lower GPx activity in SNPc	[7]
6	Sprague–Dawley rats	Intraperitoneal, 10 mg per kg (once a week for 4 weeks)	Reduced GPx activity in midbrain	[142]
7	Human	Not determined	Reduced CAT activity in SNPc and blood	[105]
8	Drosophila	Orally, 20 mM for 24 h	∼50% reduction in SOD activity in brain	[107, 136]
9	Drosophila	Orally, 20 mM for 48 h	∼2.2‐folds SOD↑ in head, ∼1.6 folds CAT↑ in brain	[108]
10	Human	Not determined	SOD↑ in SNPc and RBCs	[7]

The depletion of this major antioxidant could be due to an enhanced release of GSH disulfide (GSSG) principally from the glial cells [20]. The level of GSH within the cell is balanced at an equilibrium between GSH and GSSG [134]. But oxidative stress occurs when the equilibrium is disturbed and shifts in favor of GSSG [111]. Antioxidant defense can also be manifested by other antioxidant enzymes, which include superoxide dismutase(SOD), catalase (CAT), and GSH peroxidase (GPx) [135, 136]. The results suggest that PQ induces oxidative damage by decreasing the antioxidant defense [71]. Enhanced activities of SOD and CAT could be a defense mechanism against high concentrations of free radicals in PQ‐treated models [24].

#### 6.3.6 Paraquat and Nitrosative Stress

There are a number of studies that support that RNA has a basic role in the modulation of nitrosative stress. The fast interaction of nitric oxide and O_2_^.^ radicals leads to the formation of a byproduct, ONOO^−^, as shown in Figure 5, which leads to the generation of RNS [111, 143]. The ONOO stabilizes itself by transferring the ─NO_2_ group to tyrosine residues in proteins that constitute the neuronal cytoskeleton [144]. This results in the formation of three structural alterations in proteins that subsequently cause the death of dopaminergic neurons. 3‐nitrotyrosine is famous as one of the supposed oxidative and nitrosative stress markers related to many clinical diseases and central nervous system diseases, including PD. Great amounts of 3‐nitrotyrosine and nitroalbumin were found in the serum as well as CSF of individuals with PD in early stages. The researchers indicated that nitro‐synuclein was also detected in serum but not in CSF [73]. In GCL knockout mice, PQ (10 mg/Kg, subcutaneous, twice a week for 3 weeks) increased the level of 3‐nitrotyrosine markedly relative to their wild‐type analog. Moreover, PQ (20 mM, p.o., 24 h) has been demonstrated to increase ONOO^−^ concentration in Drosophila fly brain by 2.6‐fold [3]. Therefore, oxidative and nitrosative stress are the two major causes of paraquat‐induced neurotoxicity.

### 6.4 PQ and ER Stress

The disruption of ER integrity leads to ER stress in the cell in response to improperly folded proteins. This disruption also causes alterations in Ca^2+^ metabolism and redox balance of the organelle [41]. ER stress frequently occurs in microglia [145]. Stress‐induced ROS, along with inflammatory factors, may disrupt the usual process of regulatory pathways, including Wnt/beta‐catenin and LXR [146]. In studying the mechanism of PQ's action on the nervous system, certain researchers discovered that PQ can interfere with normal hippocampal structure, impair neural signal transmission, and even impair hippocampal memory and cognitive function [147]. Additional studies have revealed that PQ has the ability to inhibit the signal transmission of estrogen through the influence of the expression of estrogen receptors in hippocampal nerves and ultimately distorts the activity of the endoplasmic reticulum, which results in the death of hippocampal neurons [148]. Adult mice were exposed to 20 mg per kg of PQ, and it was discovered that the two kinds of neural stem cells, such as SGZ and SVZ, were killed [149]. Simultaneously, there is an increase in the number of inflammatory factors such as IL‐1β, IL‐6, and tumor necrosis factors has been observed in these two neuronal stem cells [69, 150]. These inflammatory factors are usually generated following the stress reaction of the ER of microglia, which implies that PQ can cause neuroinflammation [149]. It also further proved an association between the ER of microglia and PQ, yet the particular correlation pathway should be investigated in detail.

### 6.5 PQ and Long‐Lasting Dopamine Overflow and Reduction in Dopamine Production

A key mechanism of neurodegeneration in several neurological disorders is NMDA receptor‐mediated excitotoxicity correlating with Ca^2+^ influx into the cells [24]. It is also becoming apparent that excitotoxic damage is one of the key factors that contribute to the progression of DA neuronal loss in PD. In vivo experiments on PQ‐based toxicity in the striatum guided that PQ activates glutamate release, triggering excitotoxicity due to RNS [76]. Nitric oxide synthase (NOS) expressing neurons become activated, and following this, the NMDA receptor channels undergo evoked depolarization and allow Ca^2+^ to enter the cells through non‐NMDA receptor channels. It was also observed that the increase in extracellular glutamate is dose‐dependent on PQ [24]. The given phenomenon was detected soon after PQ administration. The entry of Ca^2+^ into the neuronal cells induces the mobilization of the Ca^2+^‐dependent intracellular processes, such as the stimulation of NOS in neurons.

NO produced by nitric oxide synthase diffuses to the terminals of dopaminergic neurons where it is believed to contribute significantly to excitotoxicity, likely by reacting with O_2_^−^ generated by the redox cycle of PQ to form peroxynitrite (ONOO^−^) [143] as shown in Figure 5. The proteins, lipids, DNA, and RNA can be oxidized by peroxynitrite, a lipid‐permeable ion, having a broader range of chemical targets as compared to NO [75, 117]. It suppresses the activity of manganese superoxide dismutase, which may result in the elevated production of O^2−^ and ONOO^−^. Also, ONOO^−^ is a good inhibitor of the mitochondrial ETC, which reduces ATP generation [116]. Second, ONOO^−^ breaks strands of DNA and blocks DNA ligase, a factor that raises the breakage of strands in the DNA.

Interestingly, treatment with glutamate receptor antagonists, an NOS inhibitor, as well as the inhibitor of monoamine oxidase (L‐deprenyl) blocked long‐duration release of dopamine and subsequent dopaminergic neuronal cell death, providing direct evidence that chronic exposure to low doses of PQ results in increased susceptibility of the dopaminergic neurons in the nigrostriatal dopamine system through the excitotoxic pathway [73]. A paper on a PC12 cell model demonstrated that the rate‐limiting enzyme in the production of dopamine, which is tyrosine hydroxylase (TH), is preferentially nitrated after ONOO^−^ exposure [45, 140]. Basically, tyrosine residues are nitrated in TH, leading to the loss of enzymatic activity. The loss of TH activity is accompanied by a decrease in the level of DA; tyrosine nitration is the process that mediates this decrease in the striatum of mice. These findings reveal that the process of tyrosine nitration induces inactivation of TH and its following incapacity to produce DA [151]. So, there is a significant role of PQ cytotoxicity on dopaminergic terminals by glutamate‐induced RNS‐mediated cytotoxicity.

### 6.6 PQ and Disruption of DA Metabolism and Neurotoxicity

PQ causes neurotoxicity by interfering with the metabolism of DA by using various pathways [7]. It can decrease the amounts of major metabolites of dopamine, including homovanillic acid (HVA), DOPAC, and 3‐methoxytyramine (3‐MT), as well as reduce their ratios, specifically HVA:DA, DOPAC: DA, and 3‐MT: DA [73]. This interference bears a strong association with PQ^•+^, which serves as a DAT substrate. In microglia, NADPH oxidase reduces PQ^2+^ to PQ^•+^, as shown in Figure 5. This is followed by the accumulation of PQ^•+^ in the dopaminergic neurons that creates oxidative stress, cytotoxicity, and other complications in DA metabolism [102, 152]. In particular, it has been demonstrated that compromised DAT activity has been associated with the reduction of PQ^•+^‐stimulated neurotoxicity, further illustrating the significant role of the DAT in regulating PQ [153].

**FIGURE 7:**
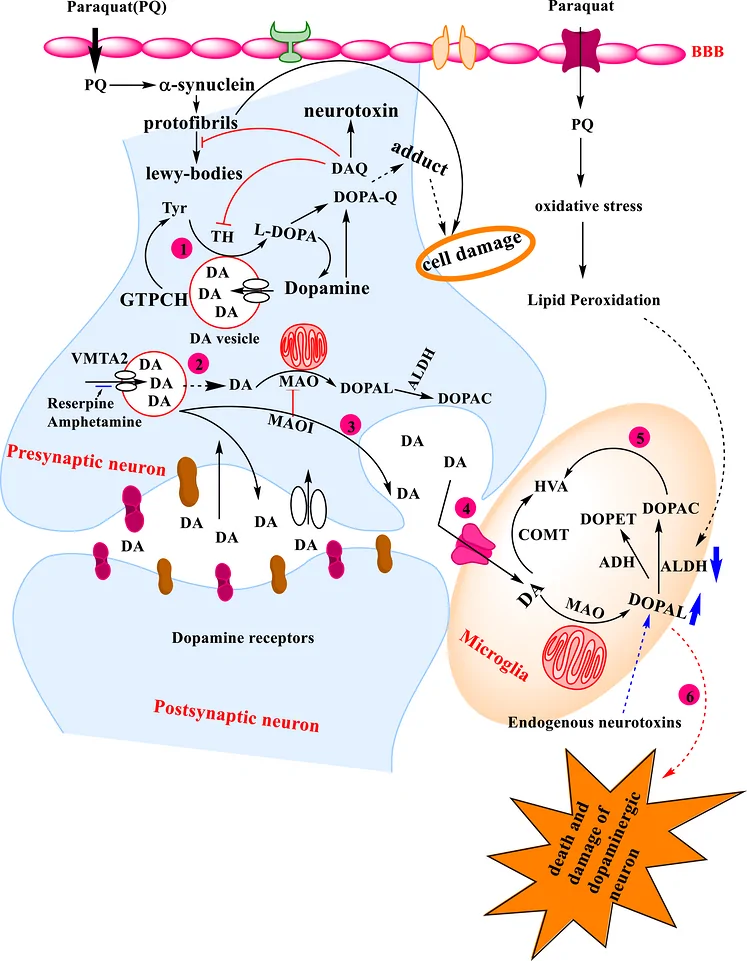
Neuronal DA metabolism.

Besides DAT, PQ^•+^ is also a substrate of the organic cation transporter 3 (Oct3) that is abundantly present in non‐dopaminergic cells in the nigrostriatal regions [130]. Paraquat also raises levels of cytosolic DA through stimulation of tyrosine hydroxylase and inhibition of monoamine oxidase (MAO) [111, 154], as shown in Figure 7. This causes a buildup of dopamine and the development of harmful quinoproteins. Furthermore, PQ also decreases the expression of VMAT2, which leads to impairment of dopamine sequestration into vesicles and enhances cytosolic concentration of dopamine [155], as shown in Figure 7. In spite of the increased TH activity, there is no change in catechol‐O‐methyltransferase (COMT) levels [53, 140]. The higher level of cytosolic DA leads to the disruption of DA metabolism, which ultimately enhances the risk of oxidation of DA and generation of ROS. Though PQ alone produces ROS, its combination with dopamine induces an enormous elevation of ROS [154]. Precisely, it would mean that the paraquat's oxidized form PQ^2+^ stimulates the brain mitochondrial generation of hydrogen peroxide (H_2_O_2_), and the complex III and MMP contribute substantially to this process [28].

Production of DA by the synergistic action of TH and aromatic amino acid decarboxylase (AADC) in the neurite of dopaminergic neurons and its transportation into synaptic vesicles by VMAT2.

1. Leakage of DA from vesicles and its deamination by MAO.
2. Upon neuronal excitation, the release of DA into the synaptic cleft for the transduction of the signal.
3. Termination of DA signaling by reuptake of DA into presynaptic neurons or into other surrounding cells and its cleavage by MAO, ALDH, and COMT.
4. DOPAC and HVA are the major degradation products of DA. Construction of NM in cytoplasmic vesicles out of the products of oxidation of DA and other constituents, and its ability to chelate iron. Oxidation of DA or DOPA to their respective reactive quinones that go on to react with a host of partially neurotoxic compounds and protein adducts.
5. Neurodegeneration and cell death due to these toxins.

### 6.7 PQ and Neuroinflammation

Neuroinflammation, which is one of the most comprehensive immune responses in the brain, is an essential defense mechanism in the brain, mediated mainly by astrocytes and microglia [58, 156]. However, under the stimulation of chronic actions that are either triggered by genetic factors or by exposure to environmental toxins such as pesticides, this protective response of the brain turns maladaptive, leading to the establishment of a pro‐inflammatory state that stimulates neurotoxicity and gradual degeneration of neurons [157]. Neurotoxicity caused by PQ is a good demonstration of how exposure to pesticides elicits a pathological neuroinflammation due to chronic exposure [158]. Exposure to paraquat also led to severe neuroinflammatory reactions in the frontal cortex, SN, and hippocampus of rodent models that were marked by the elevation of several pro‐inflammatory factors, such as TNF‐α along with IL‐1β, that have a pronounced role in the pathogenesis of PD [159]. The SNpc is especially susceptible, where massive activation of microglia in the brain is associated with continuous and progressive dopaminergic neuronal cell death and further strengthens the relationship between the chronically activated glial state and neurodegeneration in PD [156, 160].

In vivo studies also demonstrated the indirect action of PQ on microglial activation [28]. In microglial cells (BV2), exposure to PQ causes morphological alterations indicative of an activated phenotype, elevation of IL‐1β mRNA and TNF‐α [161]. It also enhances chaperonin protein expression and increases the expression of toll‐like receptor 4 (TLR4), which implies the stimulation of the innate immune signaling pathways that centrally participate in inflammation as well as glial‐mediated neurotoxicity [160]. Furthermore, two doses of PQ given to mice have produced enhanced activation of microglia in SNpc that progressed over time, leading to apoptosis of dopaminergic neurons [75]. Activation of NADPH oxidase, which is a hallmark of oxidative stress in microglia, leads to the production of ROS, which further enhances the susceptibility of dopaminergic neurons to PQ‐induced damage [102, 162].

Astrocytes are major contributors to PQ‐induced neuroinflammation [31]. During early differentiation in cell cultures of 3D whole brain of rat, low‐dose PQ induces delayed microglial activation that is preceded by astrogliosis [161]. This biphasic glial reaction is also associated with an initial increase in pro‐inflammatory cytokines such as IL‐1β and IL‐6 that is linked with the decrement of the anti‐inflammatory IL‐4, indicating a transition toward pro‐inflammatory status [163]. Interestingly, astrocytic activation continues to occur even after the removal of PQ, and proteomic studies show that post‐withdrawal astrocyte secretome alterations are long‐lasting. A major increase in microglial activation at 20 days post‐exposure is characterized by an increase in CD86, IB4 (isolectin I‐B4), and ITGAM (integrin alpha M), with TNFα levels still elevated, indicating the possibility of chronic microglial dysfunction driving neuroinflammation even after the pesticide is cleared [156, 164]. This persistent glial stimulation is an important way in which pesticide exposure will lead to gradual neurodegeneration, eventually leading to the loss of neuronal survival and rapid onset of PD [71].

### 6.8 PQ and Changes in Genes and Proteins

PQ enhances the expression of inflammatory genes while suppressing the expression of anti‐inflammatory genes [165]. It has been demonstrated that there may be varying neurotoxicity of PQ in the brains of close family members because of different genetic susceptibility, primarily due to the disparity in ferritin gene expression and the loss of the staining of TH‐positive neurons in the ventral midbrain [166]. The greater the concentration of iron, the more severe the nerve damage [75]. PQ also stimulates oxidative stress [151] and leads to nerve degeneration through the regulation of the innate immune response of genes, including stimulation of transcription factor NF‐kβ ARISE and JNK signaling factors in the Drosophila model [71, 130]. It also suppresses the production of antimicrobial peptides in the Toll and IMD pathways, thereby enhancing the chances of bacterial infection [167]. The important genes and alterations induced by PQ and their clinical manifestations are demonstrated in Table 4. The PQ‐induced PD can be influenced by interferon gamma (IFNγ). Knockout of IFN‐γ genes can reduce PQ‐stimulated inflammation and neurotoxic effects.

**TABLE 4:** Modulations in genes and proteins by PQ and its effect in causing neurodegeneration.

Sr No	Genes/proteins	Mechanism of action	Clinical manifestations	References
1	TrX1and γ‐GCS↑	Damage to nerve cells via oxidation	Neurodegeneration	[71, 168]
2	IFN‐γ↑	Stimulates inflammatory response	Inflammation of neurons	[169]
3	oct 3 ↓	Promotes oxidative stress and blocks the nervus striatum pathway	Damage to nerves	[71, 154]
4	GSTα, LRRK↑, GSTPi, and MDR1	Promote cellular oxidation	Oxidative stress	[170, 171]
5	Gdf3, Oc4, and Sox1↓	Reduces the release of vascular endothelial factors, blocks differentiation and proliferation, stimulates the apoptosis of stem cells of neurons	Damage to nerves	[172, 173]

### 6.9 Activation of Apoptotic Signaling Pathways

Apoptosis is the major mechanism of cell death of neurons in PD [64]. The event of cell death is marked by morphological alterations of the cells, which include shrinkage of the cell, condensation of chromatin material, blebbing of the plasma membrane, and fragmentation of the nucleus [130]. In development and aging, apoptosis has an important role in many physiological processes to either preserve or destroy unwanted and redundant populations in tissues. Apoptosis also has a vital function in the formation and maturation of neurons, specifically to provide shaping of neurons and the nervous system and development of proper neuronal circuitry. The initiation of this programmed cell death, apoptosis, is a very well‐regulated process that is triggered by an intracellular proteolytic cascade of proteins called caspases. The caspases are also produced in the cells as procaspases and are activated by proteolytic cleavage at the aspartic acid residues via a second member of the caspase family for further amplification of the proteolytic cascade [7].

Apoptotic caspases have been classified into initiator caspases (caspase‐8,9) that activate the apoptotic machinery and the executioner caspases like caspase 3, 6, and 7, which destroy the components of the cell by mass proteolysis [7, 158]. BCL‐2 family of intracellular proteins is the major upstream regulator of apoptosis, and it regulates apoptosis through direct physical protein‐protein interaction, which either promotes or inhibits mitochondrial outer membrane permeabilization (MOMP) [174]. This family of proteins is characterized into three basic groups: (i) anti‐apoptotic proteins, which include BCL‐2 along with myeloid cell leukemia‐1 (MCL‐1) and BCL‐XL, (ii) pro‐apoptotic (pore‐forming proteins), which include BAK and BAX, (iii) pro‐apoptotic proteins (BH3‐ only) that consist of Noxa, BAD, BNIP3, and so forth. The regulation of this balance between pro‐ and anti‐apoptotic members of the BCL‐2 family is critical for deciding the fate of the cell as to whether it will survive or die [125]. BH3‐only proteins attach themselves to the outer membrane of the mitochondria when the cells have been committed to apoptosis, and this enhances the binding affinity of BAK and BAX. This will lead to oligomerization and insertion of BAK and BAX into the bilayer of mitochondria to create pores or MOMP [175]. This is followed by the secretion of pro‐apoptotic proteins into the intermembrane space of the mitochondria; these proteins include cytochrome c, Smac/DIABLO, and apoptosis‐stimulating factors, and they enter the cytosol and recruit and activate the caspases [158].

**FIGURE 8:**
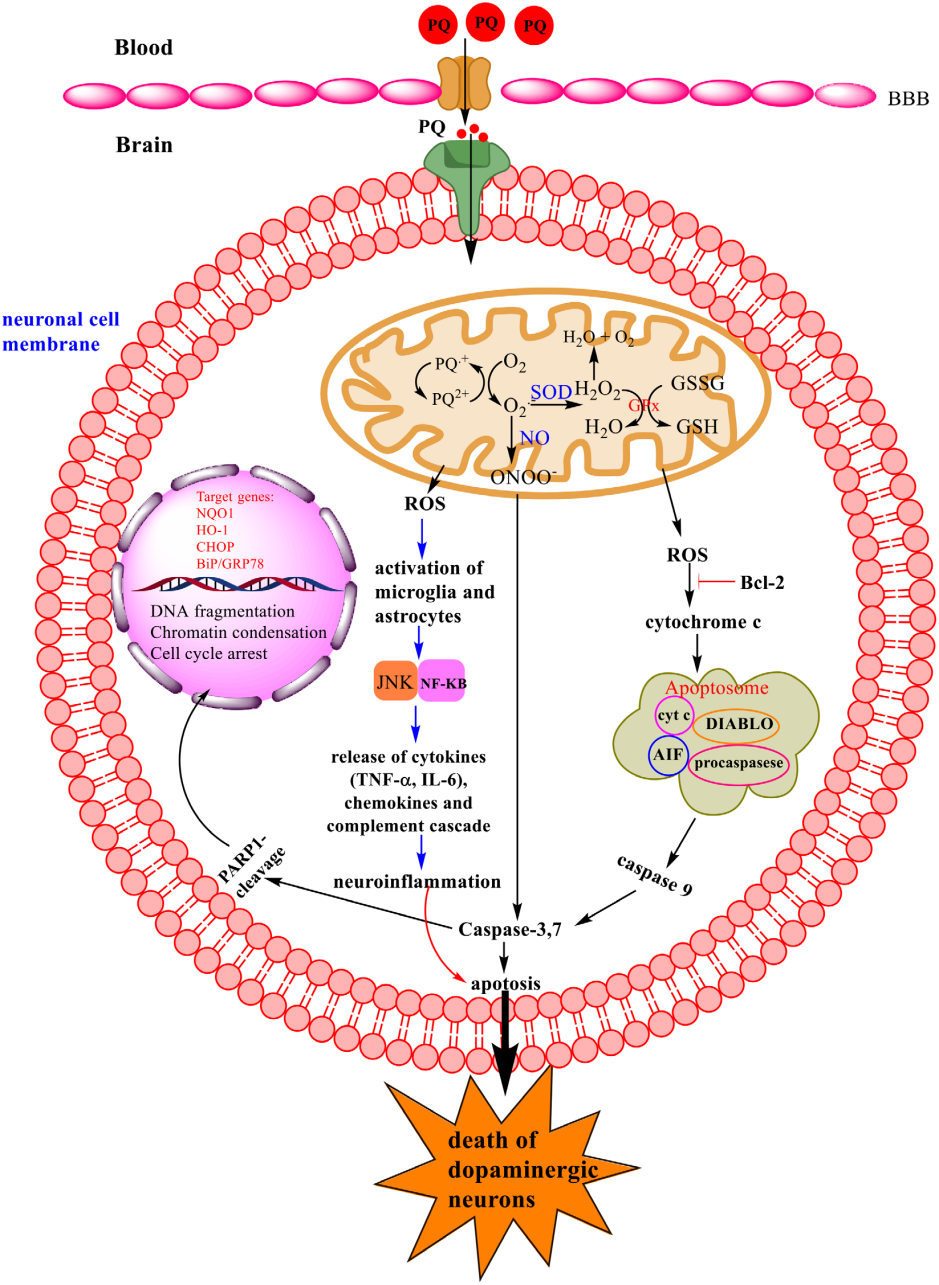
Oxidative stress, neuroinflammation and apoptosis of dopaminergic neurons.

Different extrinsic and intrinsic pathological signals may activate apoptosis; the most important ones include ROS and RNS in the case of PQ exposure [176]. There are innumerable in vitro and murine models that have shown neurotoxicity due to exposure to PQ mediated by ROS‐induced apoptosis [151]. Moreover, PQ neurotoxicity was found to be mediated through a BAK‐ dependent pathway that includes BH3‐only proteins, BNIP3, and Noxa, which are upstream of BAK [53]. BAK is expressed constitutively on the surface of mitochondria, and it is suppressed by pro‐survival proteins, which include BCL‐2, MCL‐1, and BCL‐XL [67]. Noxa/MCL‐1 and BNIP3/BCL‐2/BCL‐XL binding leads to the disinhibition of the BAK pathway as well as activation of MOMPS. A research study has shown that the treatment of SK‐NSH cells with PQ resulted in the upregulation of the expression of mRNA and proteins of BAK, BNIP3, and Noxa, as well as the levels of cytochrome c and cleaved caspase‐3, which implies the initiation of the apoptotic cascade [177, 178]. Besides, cytochrome c and PQ were shown to release other pro‐apoptotic intermembrane space proteins, including HtrA2/Omi and Smac/DIABLO, but not AIF and endonuclease G as shown in Figure 8 [179]. The major pharmacological impacts of PQ are shown in Table 5, which are dose‐dependent processes in different in vitro, in vivo, and in silico models.

**TABLE 5:** Pharmacological impacts of PQ.

Type of study	Model type (cell/organism)	Dose of PQ	Route used	Duration of exposure	Effects on body	References
In vitro	Human neuroblastoma cells (SH‐SY5Y cells)	300µM	Not applicable	24 h	HtrA2↑, cytochrome c↑, cyclin D1↓, β‐catenin↑, cleaved PARP↑, DIABLO, BAK↑, p‐GSK‐3α/β↑, and cleaved caspase 3↑	[151, 180, 181]
In vitro	Embryonic fibroblasts of mouse (NIH‐3T3)	100–1000 µM	Not applicable	24 h	DNA fragmentation↑, ERK1/2↑, cytochrome c↑, and p‐EIk1↑	[130]
In vitro	Human neuroblastoma cells (SH‐SY5Y) overexpression of wild‐type ASK1	100 µM	Not applicable	24 h	ER stress↑ and autophagy↑	[7, 97]
In vitro	Brain neuroblasts (E18 cells) of rat	25 µM	Not applicable	24 h	p‐AKT↑, p‐JNK1/2↑, and p‐ERK1/2↑	[97]
In vitro	Neuroblastoma cells (SH‐SY5Y cells)	300 µM	Not applicable	24 h	p‐AKT↑, p‐IGF1R↓, caspase‐3↑, cytochrome c↑, BAD↑, p‐c‐Jun↑, p‐JNK↑, TUNEL‐+ve cells↑, p‐PI3K↑, PARP↑, caspase 9↑, BAX, BCL‐2 ratio↑, p‐p53↑, p‐ERK↑, and p‐p38↑	[97, 182]
In vitro	Human neuroblastoma cells (SH‐SY5Y)	25–750 µM	Not applicable	24 h	p‐c‐Jun↑, p‐p38↑, p‐ASK1ser967↓, ASK1↑, p‐JNK, p‐ASK1thr845↑, p‐ASKser83↓, and p‐MKK4/7↑	[97, 133]
In vivo	Male Drosophila melanogaster (w^1118^)	10–20 mM	Oral	12–24 h	TUNEL positive cells↑, p‐JNK↑, cleaved caspase‐3↑, and pFOXO/FOXO↑,	[62, 183]
In vivo	Male Drosophila melanogaster (Oregon‐R‐P2)	20 mM	Oral	48 h	Nrf2↑, p‐JNK↑, and cleaved caspase‐3↑	[108]
In vivo	Male mice with a mixed C57BL/6 **×** 129S background (2–3 months)	10 mg/kg	intraperitoneal	twice in a week for 6 weeks	Autophagic flux↑, p‐GSK‐3β↑, α‐synuclein↑, mTOR↑, and p‐tau↑	[18, 28, 184]
Integrated in vivo + in silico (RNA‐seq, pathway analysis, and network analysis)	Mouse ventral midbrain + striatum transcriptomic model	10 mg/Kg for mice + not specified for computational analysis	Intraperitoneal for mice + not applicable for computational analysis	7 days for mice + not specified for computational analysis	Altered PD‐related signaling pathways, such as PPAR, Wnt/β catenin, GPCR, TGF‐β, axonal guidance, indicating overlap of pesticide exposure and mechanisms of PD	[185]
In silico (bio physical/computational protein‐ interaction analysis)	α‐Synuclein protein interaction model	Not applicable	Not applicable	Not specified	PQ was found to interact with α‐synuclein and potentially induce conformational changes associated with α‐synuclein misfolding and aggregation	[186]
Single‐cell transcriptomics (scRNA‐seq, functional enrichment, and trajectory analysis)	Single‐cell RNA‐sequencing mice model	Not specified	Not applicable	Not specified	Disclosed cellular heterogeneity and alterations in glutamatergic neuronal groups, indicating the involvement of neuronal dysfunction in PQ‐induced PD, exhibiting depressive‐like pathology	[187]
AOP construction and molecular stimulation docking	Toxicology + in silico AOP model	Not specified in computational analysis	Not applicable	Not specified	Activated complement C1q‐related neuro‐immune pathways, which result in microglia phagocytosis, synaptic loss, neuro‐inflammation, and cognitive dysfunction	[188]
Single‐cell transcriptomics (sc‐RNA‐seq integration, risk enrichment and cell‐type analysis	Brain single‐cell transcriptomic model	Not specified in computational analysis	Not applicable	Not specified	Determined pathological changes of astrocytes and possible hub genes involved in PQ‐induced PD‐MDD comorbidity	[189]
In vitro + bioinformatics + docking (Reactome pathway mapping and molecular docking)	SH‐SY5Y PQ model; NPS/NPSR1 axis	100 µM	Not specified	24 h	Neuropeptide S exhibited anti‐toxic effects against PQ‐induced toxicity via regulation of GPCR signaling pathway, ATP, dopamine, ERK1/2, Nrf2, and apoptosis‐related pathways	[29]
Network toxicology, sc‐RNA‐seq, and pathway analysis	Microglia‐astrocyte interaction model	Not specified in computational analysis	Not applicable	Not specified	Stimulated microglia helped in astrocyte phenotypic polarization, neuro‐inflammation, and dysregulation of the PI3K/AKT pathway	[184]
Network toxicology + experimental validation	Network toxicology and microglia model	Not specified in computational analysis	Not applicable	Not specified	Paraquat selectively interacts with microglia and stimulates neuroinflammatory changes with glycolytic reprogramming, which leads to the destruction of dopaminergic neurons	[190]

## 7 Synergistic Toxicity of PQ and Maneb

Although examination of the impacts of a single pesticide has led to invaluable findings in understanding the pathology and molecular pathways of PD, there is an increased appreciation of the need to study the effects of multiple pesticides that are used in the same agricultural areas and crops [54]. In light of the high occurrence of various pesticide residues in food products, it is highly imperative to develop models that capture this aspect in order to enable a more in‐depth perception of the causes of PD [191]. PQ and maneb are some of the most commonly used pesticides in the production of crops [66]. In mouse models in which low doses of PQ + maneb had been administered, locomotor deficits were observed due to the decreased levels of DAT and HT proteins in the striatum, accompanied by the loss of dopaminergic neurons and the development of reactive gliosis [92, 133]. Furthermore, a one‐dose administration of MPTP (15 mg/kg), which did not show any significant impact on control mice, potentiated the susceptibility of the nigrostriatal system of the mice that had previous exposure to maneb, PQ, or both. In the case of PQ + maneb exposure to double mutant mice (A30P + A53T), the exaggerated decline in levels of dopamine was strongly preceded by the loss of dopaminergic neuronal cells [53]. Nevertheless, although the dopamine levels in mice that express αsynuclein treated with both pesticides also declined, the reduction was not as sharp as in the case of the double mutant. This pattern was also detected with respect to the DOPAC, DA, and HVA turnover levels [54, 192].

Although not all studies on PD models observed of α‐synuclein aggregation, it is essential to understand the factors that promote it. A research study on mice has noted that although the synergistic toxicity of PQ and maneb is extremely dangerous, it did not result in significant elevation in α‐synuclein levels or elevated 26S proteolytic activity [193]. However, PQ‐ and maneb‐treated mice had an elevated mammalian target of mTOR [18], rapamycin, reduced autophagic flux, and upregulated HSC70, heat shock proteins 70 and 90 (HSP‐70/90) [194], suggesting failure of autophagy but not CMA, despite higher levels of autophagic proteins including ATG12 and Beclin‐1 [53, 193]. However, α‐synuclein pathology is central to PD, as demonstrated by many mutations in the α‐synuclein gene [195], oxidative stress, post‐translational alterations, and agrochemical exposure. The detailed mechanism by which these factors cause α‐synuclein aggregation is not fully explained yet. As one of the studies has revealed that the α‐synuclein radicals’ formation could be the first step in the aggregation of this protein in neurons of a mouse model that had been exposed to PQ + maneb [196].

## 8 Conclusion

This review has revealed the association between PQ and PD as a potent cause of oxidative stress, a condition that leads to ROS generation. The data reviewed in this article suggest that PQ causes dopaminergic neuronal injury via multiple related pathological pathways rather than through a single mechanism. The numerous biochemical pathways include oxidative injury, ER stress, inactivation or inhibition of TH, mitochondrial dysfunction, alterations in dopamine catabolism, and reduction in the neurotrophic factor BDNF, leading to the death of dopaminergic neurons located in the SNpc. These mechanistic pathways seem to amplify each other, leading to a vicious cycle of cellular stress and neuronal vulnerability. The dopaminergic neurons of the SNpc are particularly vulnerable to this toxicity due to their high oxidative stress load, reliance on mitochondrial activity, and sensitivity to inflammatory and proteostatic dysfunction. Overall results indicate that PQ exposure may drive parkinsonian pathology by affecting pathways involved in redox balance, mitochondrial function, protein aggregation, and neuronal survival pathways. Therefore, the models (in silico, in vitro, and in vivo) of PQ, directly or indirectly involved in the pathogenesis of PD under the enhanced state of oxidative stress, may give us a huge picture to establish innovative therapeutic approaches in the near future. The generation of ROS and RNS like O2 •^−^ and ONOO^−^ through redox cycling of PQ has been proposed extensively to be the most important mechanism of oxidative stress leading to the apoptosis of dopaminergic neurons. We have identified consistency in studies that examine cellular processes and in vivo organisms, and human populations, within the context of the pathogenesis of PD. The pathophysiological mechanisms with the use of laboratory animals and signaling cascades that we have discussed in this paper determine the emergence of characteristics of PD after exposure to PQ giving us imperative evidence to extend the study and eliminate the issue. But it would be more appropriate to discuss paraquat as a component of the syndrome of “pesticide‐associated Parkinsonism and Parkinsonian neurodegeneration,” rather than as a confirmed cause of idiopathic PD. In summary, this review highlights the multi‐faceted nature of the neurotoxic effects of paraquat, involving multiple pathways of oxidative stress, mitochondrial dysfunction, ER stress, neuroinflammation, and cell‐death pathways leading to dopaminergic neuronal damage.

## 9 Current Challenges and Future Perspectives

The physiological science with laboratory animals, the biochemical theories we have discussed in this paper and the etiological studies that have found a high risk of developing PD after exposure to PQ, are sufficient and necessary evidence to guide further research and resolve this problem. Nevertheless, the environmental context should be investigated in the laboratory. We are suggesting the extension of laboratory investigations to reflect the environmental context and to test models of animals, including captured wild animals and farm animals that have been to exposed to PQ in the environment, where PD prevalence has already been observed alongsided the simultaneous study of the human population. Moreover, a population‐based strategy is needed where genetics, phenotype expression, and ecotoxicology should be studied with the anticipation of regional variability in the expression of PD phenotype. Cultural differences should also be considered, including variations in safety standards for wearing appropriate PPE. Understanding the complete degree, rate, and concentration of PQ exposure via various pathways will enhance experimental practices with regard to this elusive dilemma.

## Authors Contributions

S.R. wrote and edited the manuscript and did the software work. S.A. edited and evaluated the manuscript. M.S. edited and evaluated the manuscript. S.A. Proofread and edited the manuscript. A.I. Proofread and edited the manuscript. R.A. Proofread and edited the manuscript. R.B. Proofread and edited the manuscript.

## Funding

The authors have nothing to report.

## Conflicts of Interest

The authors declare no conflicts of interest.

## Data Availability

Data sharing does not apply to this article, as no datasets were generated or analyzed during the current study.

## References

[1] J. Zhu , Y. Cui , J. Zhang , et al., “Temporal Trends in the Prevalence of Parkinson's Disease From 1980 to 2023: A Systematic Review and Meta‐Analysis,” Lancet Healthy Longevity 5, no. 7 (2024): e464–e479, 10.1016/S2666-7568(24)00094-1. 38945129

[2] F. Bosak , V. Baradaran Rahimi , B. Sobhani , M. M. Dabbaghi , M. Soukhtanloo , F. Zahedi Avval , and V. R. Askari , “Evaluation of the Protective Effects of Noscapine on Paraquat‐Induced Parkinson's Disease in Rats,” Molecular Neurobiology 62 (2025): 11848–11876, 10.1007/s12035-025-05000-6. 40327305

[3] P. Sharma and P. Mittal , “Paraquat (Herbicide) as a Cause of Parkinson's Disease,” Parkinsonism & Related Disorders 119 (2024): 105932, 10.1016/j.parkreldis.2023.105932. 38008593

[4] B. Vellingiri , M. Chandrasekhar , S. Sri Sabari , et al., “Neurotoxicity of Pesticides–A Link to Neurodegeneration,” Ecotoxicology and Environmental Safety 243 (2022): 113972, 10.1016/j.ecoenv.2022.113972. 36029574

[5] K. B. Bhattacharyya , “Hallmarks of Clinical Aspects of Parkinson's Disease Through Centuries,” International Review of Neurobiology 132 (2017): 1–23, 10.1016/bs.irn.2017.01.003. 28554405

[6] H. Chen , “Are we Ready for a Potential Increase in Parkinson Incidence?,” JAMA Neurology 73, no. 8 (2016): 919, 10.1001/jamaneurol.2016.1599. 27322659

[7] W. Z. C. See , R. Naidu , and K. S. Tang , “Cellular and Molecular Events Leading to Paraquat‐Induced Apoptosis: Mechanistic Insights Into Parkinson's Disease Pathophysiology,” Molecular Neurobiology 59, no. 6 (2022): 3353–3369, 10.1007/s12035-022-02799-2. 35306641 PMC9148284

[8] A. B. R. Leite Silva , R. W. Gonçalves De Oliveira , G. P. Diógenes , et al., “Premotor, Nonmotor and Motor Symptoms of Parkinson's Disease: A New Clinical State of the Art,” Ageing Research Reviews 84 (2023): 101834, 10.1016/j.arr.2022.101834. 36581178

[9] Y. Ben‐Shlomo , S. Darweesh , J. Llibre‐Guerra , C. Marras , M. San Luciano , and C. Tanner , “The Epidemiology of Parkinson's Disease,” Lancet 403, no. 10423 (2024): 283–292, 10.1016/S0140-6736(23)01419-8. 38245248 PMC11123577

[10] E. Tolosa , A. Garrido , S. W. Scholz , and W. Poewe , “Challenges in the Diagnosis of Parkinson's Disease,” Lancet Neurology 20, no. 5 (2021): 385–397, 10.1016/S1474-4422(21)00030-2. 33894193 PMC8185633

[11] A. Chopra , A. E. Lang , G. Höglinger , and T. F. Outeiro , “Towards a Biological Diagnosis of PD,” Parkinsonism & Related Disorders 122 (2024): 106078, 10.1016/j.parkreldis.2024.106078. 38472075

[12] Z. Ou , J. Pan , S. Tang , D. Duan , D. Yu , H. Nong , and Z. Wang , “Global Trends in the Incidence, Prevalence, and Years Lived With Disability of Parkinson's Disease in 204 Countries/Territories From 1990 to 2019,” Frontiers in Public Health 9 (2021): 776847, 10.3389/fpubh.2021.776847. 34950630 PMC8688697

[13] D. T. Dexter and P. Jenner , “Parkinson Disease: From Pathology to Molecular Disease Mechanisms,” Free Radical Biology and Medicine 62 (2013): 132–144, 10.1016/j.freeradbiomed.2013.01.018. 23380027

[14] S. Y.‐Y. Pang , P. W.‐L. Ho , H.‐F. Liu , et al., “The Interplay of Aging, Genetics and Environmental Factors in the Pathogenesis of Parkinson's Disease,” Translational Neurodegeneration 8, no. 1 (2019): 23, 10.1186/s40035-019-0165-9. 31428316 PMC6696688

[15] C. Marras , J. C. Beck , J. H. Bower , et al., Parkinson's Foundation P4 Group , “Prevalence of Parkinson's Disease Across North America,” NPJ Parkinson's Disease 4, no. 1 (2018): 21, 10.1038/s41531-018-0058-0. PMC6039505 30003140

[16] S. J. McCann , “Neuroticism, Urbanization, and the State Prevalence of Parkinson's Disease in the USA,” Journal of Research in Personality 102 (2023): 104319, 10.1016/j.jrp.2022.104319.

[17] E. R. Dorsey and B. R. Bloem , “Parkinson's Disease is Predominantly an Environmental Disease,” Journal of Parkinson's Disease 14, no. 3 (2024): 451–465, 10.3233/JPD-230357. PMC11091623 38217613

[18] W. Z. C. See , R. Naidu , and K. S. Tang , “Paraquat and Parkinson's Disease: The Molecular Crosstalk of Upstream Signal Transduction Pathways Leading to Apoptosis,” Current Neuropharmacology 22, no. 1 (2024): 140–151. 36703582 10.2174/1570159X21666230126161524 PMC10716878

[19] G. Bonato , A. Antonini , F. Pistonesi , et al., “Genetic Mutations in Parkinson's Disease: Screening of a Selected Population From North‐Eastern Italy,” Neurological Sciences 46, no. 1 (2025): 165–174, 10.1007/s10072-024-07690-7. 39034353 PMC11698772

[20] S. Bastías‐Candia , J. M. Zolezzi , and N. C. Inestrosa , “Revisiting the Paraquat‐Induced Sporadic Parkinson's Disease‐Like Model,” Molecular Neurobiology 56, no. 2 (2019): 1044–1055, 10.1007/s12035-018-1148-z. 29862459

[21] M. E. Newell , A. Aravindan , A. Babbrah , and R. U. Halden , “Epigenetic Biomarkers Driven by Environmental Toxins Associated With Alzheimer's Disease, Parkinson's Disease, and Amyotrophic Lateral Sclerosis in the United States: A Systematic Review,” Toxics 13, no. 2 (2025): 114, 10.3390/toxics13020114. 39997929 PMC11860158

[22] Y. Zhang , Y. Jiang , Z. Yu , et al., “Characterizing Microglial Heterogeneity in Autophagy Impairment of Paraquat‐Induced Parkinson's Disease‐Like Neurodegeneration,” Ecotoxicology and Environmental Safety 299 (2025): 118364, 10.1016/j.ecoenv.2025.118364. 40403688

[23] S. M. Fathy , H. A. El‐Dash , and N. I. Said , “Neuroprotective Effects of Pomegranate (*Punica granatum* L.) Juice and Seed Extract in Paraquat‐Induced Mouse Model of Parkinson's Disease,” BMC Complementary Medicine and Therapies 21, no. 1 (2021): 130, 10.1186/s12906-021-03298-y. 33902532 PMC8074500

[24] N. F. Naspolini , C. E. Heinz Rieg , V. H. Cenci , D. Cattani , and A. Zamoner , “Paraquat Induces Redox Imbalance and Disrupts Glutamate and Energy Metabolism in the Hippocampus of Prepubertal Rats,” Neurotoxicology 85 (2021): 121–132, 10.1016/j.neuro.2021.05.010. 34048864

[25] L. G. Gunnarsson and L. Bodin , “Occupational Exposures and Neurodegenerative Diseases—A Systematic Literature Review and Meta‐Analyses,” International Journal of Environmental Research and Public Health 16, no. 3 (2019): 337, 10.3390/ijerph16030337. 30691095 PMC6388365

[26] D. L. Weed , “Does Paraquat Cause Parkinson's Disease? A Review of Reviews,” Neurotoxicology 86 (2021): 180–184, 10.1016/j.neuro.2021.08.006. 34400206

[27] C. Vaccari , R. El Dib , and J. L. V. de Camargo , “Paraquat and Parkinson's Disease: A Systematic Review Protocol According to the OHAT Approach for Hazard Identification,” Systematic Reviews 6, no. 1 (2017): 98, 10.1186/s13643-017-0491-x. 28506248 PMC5433017

[28] Y. T. Zhou , Y. N. Xu , X. Y. Ren , and X. F. Zhang , “Inactivation of Microglia Dampens Blood‐Brain Barrier Permeability and Loss of Dopaminergic Neurons in Paraquat‐Lesioned Mice,” Food and Chemical Toxicology 174 (2023): 113692, 10.1016/j.fct.2023.113692. 36842752

[29] F. G. Koçancı , M. Bülbül , İ. Akçalı , D. Kipmen‐Korgun , E. Çubukçu , M. A. Aslan , and A. Agar , “Neuropeptide S Protects Dopaminergic Neurons in a Paraquat‐Induced Parkinson's Model Using SH‐SY5Y Cells,” Molecular Neurobiology 63, no. 1 (2026): 416. 41627613 10.1007/s12035-025-05401-7 PMC12864285

[30] P. Xiao , S. Wu , Z. Wang , G. Shen , and X. Shi , “Biotoxicity of Paraquat to Lung Cells Mediated by Endoplasmic Reticulum‐Mitochondria Interaction,” Journal of Molecular Histology 55, no. 6 (2024): 1063–1077, 10.1007/s10735-024-10249-7. 39215928

[31] X. Yuan , Y. Tian , C. Liu , and Z. Zhang , “Environmental Factors in Parkinson's Disease: New Insights Into the Molecular Mechanisms,” Toxicology Letters 356 (2022): 1–10, 10.1016/j.toxlet.2021.12.003. 34864130

[32] A. Ascherio and M. A. Schwarzschild , “The Epidemiology of Parkinson's Disease: Risk Factors and Prevention,” Lancet Neurology 15, no. 12 (2016): 1257–1272, 10.1016/S1474-4422(16)30230-7. 27751556

[33] C. Y. Song , M. X. Feng , L. Li , P. Wang , X. Lu , and Y. Q. Lu , “ *Tripterygium wilfordii* Hook. f. Ameliorates Paraquat‐Induced Lung Injury by Reducing Oxidative Stress and Ferroptosis via Nrf2/HO‐1 Pathway,” Ecotoxicology and Environmental Safety 252 (2023): 114575, 10.1016/j.ecoenv.2023.114575. 36706526

[34] A. M. Stuart , C. N. Merfield , F. G. Horgan , et al., “Agriculture Without Paraquat is Feasible Without Loss of Productivity—Lessons Learned From Phasing Out a Highly Hazardous Herbicide,” Environmental Science and Pollution Research 30, no. 7 (2023): 16984–17008, 10.1007/s11356-022-24951-0. 36622585 PMC9928820

[35] W. Tangamornsuksan , O. Lohitnavy , R. Sruamsiri , N. Chaiyakunapruk , C. Norman Scholfield , B. Reisfeld , and M. Lohitnavy , “Paraquat Exposure and Parkinson's Disease: A Systematic Review and Meta‐Analysis,” Archives of Environmental & Occupational Health 74, no. 5 (2019): 225–238, 10.1080/19338244.2018.1492894. 30474499

[36] D. Weintraub , D. Aarsland , K. R. Chaudhuri , R. D. Dobkin , A. F. Leentjens , M. Rodriguez‐Violante , and A. Schrag , “The Neuropsychiatry of Parkinson's Disease: Advances and Challenges,” Lancet Neurology 21, no. 1 (2022): 89–102, 10.1016/S1474-4422(21)00330-6. 34942142 PMC8800169

[37] A. Bayati , R. Ayoubi , A. Aguila , et al., “Modeling Parkinson's Disease Pathology in Human Dopaminergic Neurons by Sequential Exposure to *α*‐Synuclein Fibrils and Proinflammatory Cytokines,” Nature Neuroscience 27, no. 12 (2024): 2401–2416, 10.1038/s41593-024-01775-4. 39379564

[38] S. P. Caminiti , L. Presotto , D. Baroncini , et al., “Axonal Damage and Loss of Connectivity in Nigrostriatal and Mesolimbic Dopamine Pathways in Early Parkinson's Disease,” NeuroImage: Clinical 14 (2017): 734–740, 10.1016/j.nicl.2017.03.011. 28409113 PMC5379906

[39] R. Soni , K. Mathur , and J. Shah , “An Update on New‐Age Potential Biomarkers for Parkinson's Disease,” Ageing Research Reviews 94 (2024): 102208, 10.1016/j.arr.2024.102208. 38296162

[40] F. Miraglia , A. Ricci , L. Rota , and E. Colla , “Subcellular Localization of Alpha‐Synuclein Aggregates and Their Interaction With Membranes,” Neural Regeneration Research 13, no. 7 (2018): 1136–1144. 30028312 10.4103/1673-5374.235013 PMC6065224

[41] N. S. Walter , V. Gorki , R. Bhardwaj , and P. Punnakkal , “Endoplasmic Reticulum Stress: Implications in Diseases,” Protein Journal 44, no. 2 (2025): 147–161, 10.1007/s10930-025-10264-x. 40082380

[42] U. Sengupta and R. Kayed , “Amyloid β, Tau, and *α*‐Synuclein Aggregates in the Pathogenesis, Prognosis, and Therapeutics for Neurodegenerative Diseases,” Progress in neurobiology 214 (2022): 102270, 10.1016/j.pneurobio.2022.102270. 35447272

[43] M. Funayama , K. Nishioka , Y. Li , and N. Hattori , “Molecular Genetics of Parkinson's Disease: Contributions and Global Trends,” Journal of Human Genetics 68, no. 3 (2023): 125–130, 10.1038/s10038-022-01058-5. 35821405 PMC9968657

[44] C. Blauwendraat , M. A. Nalls , and A. B. Singleton , “The Genetic Architecture of Parkinson's Disease,” Lancet Neurology 19, no. 2 (2020): 170–178, 10.1016/S1474-4422(19)30287-X. 31521533 PMC8972299

[45] R. J. Dinis‐Oliveira , F. Remião , H. Carmo , et al., “Paraquat Exposure as an Etiological Factor of Parkinson's Disease,” Neurotoxicology 27, no. 6 (2006): 1110–1122, 10.1016/j.neuro.2006.05.012. 16815551

[46] D. Belvisi , R. Pellicciari , A. Fabbrini , et al., “Risk Factors of Parkinson Disease,” Neurology 95, no. 18 (2020): e2500–e2508, 10.1212/WNL.0000000000010813. 32943485 PMC7682833

[47] S. Kab , J. Spinosi , L. Chaperon , A. Dugravot , A. Singh‐Manoux , F. Moisan , and A. Elbaz , “Agricultural Activities and the Incidence of Parkinson's Disease in the General French Population,” European Journal of Epidemiology 32, no. 3 (2017): 203–216, 10.1007/s10654-017-0229-z. 28185034

[48] A. Kouli , K. M. Torsney , and W. L. Kuan , Parkinson's Disease: Etiology, Neuropathology, and Pathogenesis (Exon Publications, 2018), 3–26.

[49] B. R. Bloem , M. S. Okun , and C. Klein , “Parkinson's Disease,” Lancet 397, no. 10291 (2021): 2284–2303, 10.1016/S0140-6736(21)00218-X. 33848468

[50] J. Segura‐Aguilar , Clinical Studies and Therapies in Parkinson's disease: Translations From Preclinical Models (Academic Press, 2021).

[51] A. Elbaz and F. Moisan , “The Scientific Bases to Consider Parkinson's Disease an Occupational Disease in Agriculture Professionals Exposed to Pesticides in France,” Journal of Epidemiology and Community Health 70, no. 4 (2016): 319–321, 10.1136/jech-2015-205455. 26507749

[52] W. Mohamed , “Exploring Environmental Factors Contributing to Parkinson's Disease in AfrAbian Populations,” Academia Biology 2, no. 4 (2024): 1–15, 10.20935/AcadBiol7442.

[53] L. Amaral , M. Martins , M. Côrte‐Real , T. F. Outeiro , S. R. Chaves , and A. Rego , “The Neurotoxicity of Pesticides: Implications for Parkinson's Disease,” Chemosphere 377 (2025): 144348, 10.1016/j.chemosphere.2025.144348. 40203643

[54] A. Dovonou , C. Bolduc , V. Soto Linan , C. Gora , M. R. Peralta Iii , and M. Lévesque , “Animal Models of Parkinson's Disease: Bridging the Gap Between Disease Hallmarks and Research Questions,” Translational neurodegeneration 12, no. 1 (2023): 36, 10.1186/s40035-023-00368-8. 37468944 PMC10354932

[55] N. Fujikake , M. Shin , and S. Shimizu , “Association Between Autophagy and Neurodegenerative Diseases,” Frontiers in Neuroscience 12 (2018): 255, 10.3389/fnins.2018.00255. 29872373 PMC5972210

[56] B. R. Pinho , S. D. Reis , R. C. Hartley , M. P. Murphy , and J. M. Oliveira , “Mitochondrial Superoxide Generation Induces a Parkinsonian Phenotype in Zebrafish and Huntingtin Aggregation in Human Cells,” Free Radical Biology and Medicine 130 (2019): 318–327, 10.1016/j.freeradbiomed.2018.10.446. 30389496 PMC6340810

[57] D. Hernandez‐Baltazar , L. M. Zavala‐Flores , and A. Villanueva‐Olivo , “The 6‐Hydroxydopamine Model and Parkinsonian Pathophysiology: Novel Findings in an Older Model,” Neurología (English Edition) 32, no. 8 (2017): 533–539, 10.1016/j.nrleng.2015.06.019. 26304655

[58] S. Mani , M. Sevanan , A. Krishnamoorthy , and S. Sekar , “A Systematic Review of Molecular Approaches That Link Mitochondrial Dysfunction and Neuroinflammation in Parkinson's Disease,” Neurological Sciences 42, no. 11 (2021): 4459–4469, 10.1007/s10072-021-05551-1. 34480241

[59] M. Lu , C. Su , C. Qiao , Y. Bian , J. Ding , and G. Hu , “Metformin Prevents Dopaminergic Neuron Death in MPTP/P‐Induced Mouse Model of Parkinson's Disease via Autophagy and Mitochondrial ROS Clearance,” International Journal of Neuropsychopharmacology 19, no. 9 (2016): pyw047, 10.1093/ijnp/pyw047. 27207919 PMC5043649

[60] T. Prorok , M. Jana , D. Patel , and K. Pahan , “Cinnamic Acid Protects the Nigrostriatum in a Mouse Model of Parkinson's Disease via Peroxisome Proliferator‐Activated Receptorα,” Neurochemical Research 44, no. 4 (2019): 751–762, 10.1007/s11064-018-02705-0. 30612307 PMC6450560

[61] C.‐C. Chao , C.‐L. Huang , J.‐J. Cheng , et al., “SRT1720 as an SIRT1 Activator for Alleviating Paraquat‐Induced Models of Parkinson's Disease,” Redox Biology 58 (2022): 102534, 10.1016/j.redox.2022.102534. 36379180 PMC9663539

[62] S. J. Chia , E. K. Tan , and Y. X. Chao , “Historical Perspective: Models of Parkinson's Disease,” International Journal of Molecular Sciences 21, no. 7 (2020): 2464, 10.3390/ijms21072464. 32252301 PMC7177377

[63] N. Naudet , E. Antier , D. Gaillard , E. Morignat , L. Lakhdar , T. Baron , and A. Bencsik , “Oral Exposure to Paraquat Triggers Earlier Expression of Phosphorylated *α*‐Synuclein in the Enteric Nervous System of A53T Mutant Human *α*‐Synuclein Transgenic Mice,” Journal of Neuropathology & Experimental Neurology 76, no. 12 (2017): 1046–1057, 10.1093/jnen/nlx092. 29040593 PMC5939863

[64] N. K. Rajawat , K. Bhardwaj , and N. Mathur , “Risk of Parkinson Disease Associated With Pesticide Exposure and Protection by Probiotics,” Materials Today: Proceedings 69 (2022): A1–A11.

[65] M. T. Baltazar , R. J. Dinis‐Oliveira , M. de Lourdes Bastos , A. M. Tsatsakis , J. A. Duarte , and F. Carvalho , “Pesticides Exposure as Etiological Factors of Parkinson's Disease and Other Neurodegenerative Diseases—A Mechanistic Approach,” Toxicology Letters 230, no. 2 (2014): 85–103, 10.1016/j.toxlet.2014.01.039. 24503016

[66] C. Liu , Z. Liu , Y. Fang , et al., “Exposure to the Environmentally Toxic Pesticide Maneb Induces Parkinson's Disease‐Like Neurotoxicity in Mice: A Combined Proteomic and Metabolomic Analysis,” Chemosphere 308 (2022): 136344, 10.1016/j.chemosphere.2022.136344. 36087732

[67] C. Liu , Z. Liu , Y. Fang , et al., “Exposure to Dithiocarbamate Fungicide Maneb In Vitro and In Vivo: Neuronal Apoptosis and Underlying Mechanisms,” Environment International 171 (2023): 107696, 10.1016/j.envint.2022.107696. 36563597

[68] S. L. Boyd , N. C. Kuhn , J. R. Patterson , et al., “Developmental Exposure to the Parkinson's Disease‐Associated Organochlorine Pesticide Dieldrin Alters Dopamine Neurotransmission in *α*‐Synuclein Pre‐Formed Fibril (PFF)‐Injected Mice,” Toxicological Sciences 196, no. 1 (2023): 99–111, 10.1093/toxsci/kfad086. 37607008 PMC10613968

[69] T. Liu , F. Zheng , L. Liu , H. Zhou , T. Shen , Y. Li , and W. Zhang , “Paraquat Disrupts the Blood–Brain Barrier by Increasing IL‐6 Expression and Oxidative Stress Through the Activation of PI3K/AKT Signaling Pathway,” Open Medicine 19, no. 1 (2024): 20241020, 10.1515/med-2024-1020. 39291284 PMC11406143

[70] R. Mesnage , A. Székács , and J. G. Zaller , “Herbicides: Brief History, Agricultural Use, and Potential Alternatives for Weed Control,” in Herbicides (Elsevier, 2021), 1–20, 10.1016/B978-0-12-823674-1.00002-X.

[71] J. Chen , Y. Su , F. Lin , M. Iqbal , K. Mehmood , H. Zhang , and D. Shi , “Effect of Paraquat on Cytotoxicity Involved in Oxidative Stress and Inflammatory Reaction: A Review of Mechanisms and Ecological Implications,” Ecotoxicology and Environmental Safety 224 (2021): 112711, 10.1016/j.ecoenv.2021.112711. 34455184

[72] Z. Liu , F. Huang , S. Zhao , L. Ma , Q. Shi , and Y. Zhou , “Homicidal Paraquat Poisoning: Poisoned While Drinking,” Journal of Forensic Sciences 67, no. 3 (2022): 1312–1319, 10.1111/1556-4029.14968. 35005788

[73] X. F. Zhang , M. Thompson , and Y. H. Xu , “Multifactorial Theory Applied to the Neurotoxicity of Paraquat and Paraquat‐Induced Mechanisms of Developing Parkinson's Disease,” Laboratory Investigation 96, no. 5 (2016): 496–507, 10.1038/labinvest.2015.161. 26829122

[74] C.‐Y. Huang , K. Sivalingam , M. A. Shibu , et al., “Induction of Autophagy by Vasicinone Protects Neural Cells From Mitochondrial Dysfunction and Attenuates Paraquat‐Mediated Parkinson's Disease Associated *α*‐Synuclein Levels,” Nutrients 12, no. 6 (2020): 1707, 10.3390/nu12061707. 32517337 PMC7352463

[75] S. H. Tan , V. Karri , N. W. R. Tay , et al., “Emerging Pathways to Neurodegeneration: Dissecting the Critical Molecular Mechanisms in Alzheimer's Disease, Parkinson's Disease,” Biomedicine & Pharmacotherapy 111 (2019): 765–777, 10.1016/j.biopha.2018.12.101. 30612001

[76] N. Sharma and S. S. A. An , “Soil to Synapse: Molecular Insights Into the Neurotoxicity of Common Gardening Chemicals in Alzheimer's and Parkinson's Disease,” International Journal of Molecular Sciences 26, no. 13 (2025): 6468, 10.3390/ijms26136468. 40650243 PMC12249792

[77] K. Prasad , B. Winnik , M. J. Thiruchelvam , B. Buckley , O. Mirochnitchenko , and E. K. Richfield , “Prolonged Toxicokinetics and Toxicodynamics of Paraquat in Mouse Brain,” Environmental Health Perspectives 115, no. 10 (2007): 1448–1453, 10.1289/ehp.9932. 17938734 PMC2022643

[78] J. R. Richardson , Y. U. Quan , T. B. Sherer , J. T. Greenamyre , and G. W. Miller , “Paraquat Neurotoxicity is Distinct From That of MPTP and Rotenone,” Toxicological Sciences 88, no. 1 (2005): 193–201, 10.1093/toxsci/kfi304. 16141438

[79] V. Vilas‐Boas , R. Silva , P. Guedes‐De‐Pinho , F. Carvalho , M. L. Bastos , and F. Remião , “RBE4 Cells are Highly Resistant to Paraquat‐Induced Cytotoxicity: Studies on Uptake and Efflux Mechanisms,” Journal of Applied Toxicology 34, no. 9 (2014): 1023–1030, 10.1002/jat.2926. 24105845

[80] A. Picca , F. Guerra , R. Calvani , R. Romano , H. J. Coelho‐Júnior , C. Bucci , and E. Marzetti , “Mitochondrial Dysfunction, Protein Misfolding and Neuroinflammation in Parkinson's Disease: Roads to Biomarker Discovery,” Biomolecules 11, no. 10 (2021): 1508, 10.3390/biom11101508. 34680141 PMC8534011

[81] R. Powers , S. Lei , A. Anandhan , et al., “Metabolic Investigations of the Molecular Mechanisms Associated With Parkinson's Disease,” Metabolites 7, no. 2 (2017): 22, 10.3390/metabo7020022. 28538683 PMC5487993

[82] A. Tozzi , M. Sciaccaluga , V. Loffredo , et al., “Dopamine‐Dependent Early Synaptic and Motor Dysfunctions Induced by *α*‐Synuclein in the Nigrostriatal Circuit,” Brain 144, no. 11 (2021): 3477–3491, 10.1093/brain/awab242. 34297092 PMC8677552

[83] G. Zhang , Y. Xia , F. Wan , et al., “New Perspectives on Roles of Alpha‐Synuclein in Parkinson's Disease,” Frontiers in Aging Neuroscience 10 (2018): 370, 10.3389/fnagi.2018.00370. 30524265 PMC6261981

[84] R. Cascella , A. Bigi , N. Cremades , and C. Cecchi , “Effects of Oligomer Toxicity, Fibril Toxicity and Fibril Spreading in Synucleinopathies,” Cellular and Molecular Life Sciences 79, no. 3 (2022): 174, 10.1007/s00018-022-04166-9. 35244787 PMC8897347

[85] J. Segura‐Aguilar , “Can We Conclude a Potential Therapeutic Action for Parkinson's Disease by Using Postmortem Tissue and a Preclinical Model Based on an Exogenous Neurotoxin?,” Cell Death & Disease 9, no. 7 (2018): 748, 10.1038/s41419-018-0798-0. 29970885 PMC6030129

[86] B. A. Hijaz and L. A. Volpicelli‐Daley , “Initiation and Propagation of *α*‐Synuclein Aggregation in the Nervous System,” Molecular Neurodegeneration 15, no. 1 (2020): 19, 10.1186/s13024-020-00368-6. 32143659 PMC7060612

[87] P. Calabresi , A. Mechelli , G. Natale , L. Volpicelli‐Daley , G. Di Lazzaro , and V. Ghiglieri , “Alpha‐Synuclein in Parkinson's Disease and Other Synucleinopathies: From Overt Neurodegeneration Back to Early Synaptic Dysfunction,” Cell Death & Disease 14, no. 3 (2023): 176, 10.1038/s41419-023-05672-9. 36859484 PMC9977911

[88] M. Gómez‐Benito , N. Granado , P. García‐Sanz , A. Michel , M. Dumoulin , and R. Moratalla , “Modeling Parkinson's Disease With the Alpha‐Synuclein Protein,” Frontiers in Pharmacology 11 (2020): 356, 10.3389/fphar.2020.00356. 32390826 PMC7191035

[89] L. D. Bernal‐Conde , R. Ramos‐Acevedo , M. A. Reyes‐Hernández , et al., “Alpha‐Synuclein Physiology and Pathology: A Perspective on Cellular Structures and Organelles,” Frontiers in Neuroscience 13 (2020): 1399, 10.3389/fnins.2019.01399. 32038126 PMC6989544

[90] G. Fusco , S. W. Chen , P. T. F. Williamson , et al., “Structural Basis of Membrane Disruption and Cellular Toxicity by *α*‐Synuclein Oligomers,” Science 358, no. 6369 (2017): 1440–1443, 10.1126/science.aan6160. 29242346

[91] K. Wakabayashi , K. Tanji , S. Odagiri , Y. Miki , F. Mori , and H. Takahashi , “The Lewy Body in Parkinson's Disease and Related Neurodegenerative Disorders,” Molecular Neurobiology 47, no. 2 (2013): 495–508, 10.1007/s12035-012-8280-y. 22622968

[92] D. Colle and M. Farina , “Oxidative Stress in Paraquat‐Induced Damage to Nervous Tissues,” in Toxicology (Academic Press, 2021), 69–78, 10.1016/B978-0-12-819092-0.00008-X.

[93] A. B. Manning‐Bog , A. L. McCormack , J. Li , V. N. Uversky , A. L. Fink , and D. A. Di Monte , “The Herbicide Paraquat Causes Up‐Regulation and Aggregation of *α*‐Synuclein in Mice,” Journal of Biological Chemistry 277, no. 3 (2002): 1641–1644, 10.1074/jbc.C100560200. 11707429

[94] L. Hou , R. Huang , F. Sun , L. Zhang , and Q. Wang , “NADPH Oxidase Regulates Paraquat and Maneb‐Induced Dopaminergic Neurodegeneration Through Ferroptosis,” Toxicology 417 (2019): 64–73, 10.1016/j.tox.2019.02.011. 30797899

[95] A. Kumar , D. Ganini , and R. P. Mason , “Role of Cytochrome *c* in *α*‐Synuclein Radical Formation: Implications of *α*‐Synuclein in Neuronal Death in Maneb‐and Paraquat‐Induced Model of Parkinson's Disease,” Molecular Neurodegeneration 11, no. 1 (2016): 70, 10.1186/s13024-016-0135-y. 27884192 PMC5122029

[96] A. Chorfa , D. Bétemps , E. Morignat , C. Lazizzera , K. Hogeveen , T. Andrieu , and T. Baron , “Specific Pesticide‐Dependent Increases in *α*‐Synuclein Levels in Human Neuroblastoma (SH‐SY5Y) and Melanoma (SK‐MEL‐2) Cell Lines,” Toxicological Sciences 133, no. 2 (2013): 289–297, 10.1093/toxsci/kft076. 23535362

[97] A. C. Nascimento , A. G. Erustes , P. Reckziegel , C. Bincoletto , R. P. Ureshino , G. J. Pereira , and S. S. Smaili , “ *α*‐Synuclein Overexpression Induces Lysosomal Dysfunction and Autophagy Impairment in Human Neuroblastoma SH‐SY5Y,” Neurochemical research 45, no. 11 (2020): 2749–2761, 10.1007/s11064-020-03126-8. 32915398

[98] S. Nuber and D. J. Selkoe , “The Parkinson‐Associated Toxin Paraquat Shifts Physiological *α*‐Synuclein Tetramers Toward Monomers That Can be Calpain‐Truncated and Form Oligomers,” American Journal of Pathology 193, no. 5 (2023): 520–531, 10.1016/j.ajpath.2023.01.010. 36773784 PMC10155269

[99] C. Su and P. Niu , “Low Doses of Single or Combined Agrichemicals Induces *α*‐Synuclein Aggregation in Nigrostriatal System of Mice Through Inhibition of Proteasomal and Autophagic Pathways,” International Journal of Clinical and Experimental Medicine 8, no. 11 (2015): 20508. 26884967 PMC4723812

[100] P. O. Fernagut , C. B. Hutson , S. M. Fleming , N. A. Tetreaut , J. Salcedo , E. Masliah , and M. Chesselet , “Behavioral and Histopathological Consequences of Paraquat Intoxication in Mice: Effects of *α*‐Synuclein Over‐Expression,” Synapse 61, no. 12 (2007): 991–1001, 10.1002/syn.20456. 17879265 PMC3097512

[101] M. B. Watson , F. Richter , S. K. Lee , et al., “Regionally‐Specific Microglial Activation in Young Mice Over‐Expressing Human Wildtype Alpha‐Synuclein,” Experimental Neurology 237, no. 2 (2012): 318–334, 10.1016/j.expneurol.2012.06.025. 22750327 PMC3443323

[102] R. E. Musgrove , M. Helwig , E. J. Bae , H. Aboutalebi , S. J. Lee , A. Ulusoy , and D. A. Di Monte , “Oxidative Stress in Vagal Neurons Promotes Parkinsonian Pathology and Intercellular *α*‐Synuclein Transfer,” Journal of Clinical Investigation 129, no. 9 (2019): 3738–3753, 10.1172/JCI127330. 31194700 PMC6715407

[103] S. Caproni , A. Di Fonzo , and C. Colosimo , “Oxidative Stress: A New Pathophysiological Pathway in Parkinson's Disease and a Potential Target of the Brain‐Sport Crosstalk,” Parkinson's Disease 2025, no. 1 (2025): 6691390, 10.1155/padi/6691390. PMC11952919 40162062

[104] P. García‐Caparrós , L. De Filippis , A. Gul , et al., “Oxidative Stress and Antioxidant Metabolism Under Adverse Environmental Conditions: A Review,” Botanical Review 87, no. 4 (2021): 421–466, 10.1007/s12229-020-09231-1.

[105] Z. Wei , X. Li , X. Li , Q. Liu , and Y. Cheng , “Oxidative Stress in Parkinson's Disease: A Systematic Review and Meta‐Analysis,” Frontiers in Molecular Neuroscience 11 (2018): 236, 10.3389/fnmol.2018.00236. 30026688 PMC6041404

[106] S. K. Ravi , R. B. Narasingappa , C. G. Joshi , T. K. Girish , and B. Vincent , “Neuroprotective Effects of *Cassia tora* Against Paraquat‐Induced Neurodegeneration: Relevance for Parkinson's Disease,” Natural Product Research 32, no. 12 (2018): 1476–1480, 10.1080/14786419.2017.1353504. 28714346

[107] A. K. Shukla , P. Pragya , H. S. Chaouhan , D. K. Patel , M. Z. Abdin , and D. Kar Chowdhuri , “A Mutation in *Drosophila methuselah* Resists Paraquat Induced Parkinson‐Like Phenotypes,” Neurobiology of Aging 35, no. 10 (2014): 2419.e1–2419.e16, 10.1016/j.neurobiolaging.2014.04.008. 24819147

[108] S. Srivastav , M. Fatima , and A. C. Mondal , “ *Bacopa monnieri* Alleviates Paraquat Induced Toxicity in *Drosophila* by Inhibiting JNK Mediated Apoptosis Through Improved Mitochondrial Function and Redox Stabilization,” Neurochemistry International 121 (2018): 98–107, 10.1016/j.neuint.2018.10.001. 30296463

[109] S. Shalikar , M. A. Mashayekhpour , S. Ebrahimi Vosta‐Kalaee , M. Abouhosseini Tabari , and A. Hajizadeh Moghaddam , “Frankincense Improves Motor Symptoms and Attenuates the Progression of Paraquat (PQ)‐Induced Parkinson's Disease in Mice by Attenuating Oxidative Stress, Inflammation, and Apoptotic Cell Death and Improving Nerve Growth Factor Gene Expression,” IBRO Neuroscience Reports 20 (2026): 276–285, 10.1016/j.ibneur.2026.02.008. 41716729 PMC12914117

[110] D. Tsikas , “Assessment of Lipid Peroxidation by Measuring Malondialdehyde (MDA) and Relatives in Biological Samples: Analytical and Biological Challenges,” Analytical Biochemistry 524 (2017): 13–30, 10.1016/j.ab.2016.10.021. 27789233

[111] E. O. Olufunmilayo , M. B. Gerke‐Duncan , and R. D. Holsinger , “Oxidative Stress and Antioxidants in Neurodegenerative Disorders,” Antioxidants 12, no. 2 (2023): 517, 10.3390/antiox12020517. 36830075 PMC9952099

[112] M. Zhao , X. Xie , Y. Ding , et al., “Glabridin Protects Against Paraquat‐Induced Acute Lung Injury by Targeting ME1 to Mitigate Oxidative Stress, Mitochondrial Dysfunction, and cGAS‐STING Activation,” Free Radical Biology and Medicine 235 (2025): 317–334, 10.1016/j.freeradbiomed.2025.04.016. 40316060

[113] P. Kowalczyk , D. Sulejczak , P. Kleczkowska , et al., “Mitochondrial Oxidative Stress—A Causative Factor and Therapeutic Target in Many Diseases,” International Journal of Molecular Sciences 22, no. 24 (2021): 13384, 10.3390/ijms222413384. 34948180 PMC8707347

[114] E. Charen and N. Harbord , “Toxicity of Herbs, Vitamins, and Supplements,” Advances in Chronic Kidney Disease 27, no. 1 (2020): 67–71, 10.1053/j.ackd.2019.08.003. 32147004

[115] F. Zheng , F. M. Gonçalves , Y. Abiko , H. Li , Y. Kumagai , and M. Aschner , “Redox Toxicology of Environmental Chemicals Causing Oxidative Stress,” Redox Biology 34 (2020): 101475, 10.1016/j.redox.2020.101475. 32336668 PMC7327986

[116] A. R. Chowdhury , J. Zielonka , B. Kalyanaraman , R. C. Hartley , M. P. Murphy , and N. G. Avadhani , “Mitochondria‐Targeted Paraquat and Metformin Mediate ROS Production to Induce Multiple Pathways of Retrograde Signaling: A Dose‐Dependent Phenomenon,” Redox Biology 36 (2020): 101606, 10.1016/j.redox.2020.101606. 32604037 PMC7327929

[117] S. Han , Y. Feng , M. Guo , et al., “Role of OCT3 and DRP1 in the Transport of Paraquat in Astrocytes: A Mouse Study,” Environmental Health Perspectives 130, no. 5 (2022): 057004, 10.1289/EHP9505. 35511227 PMC9070608

[118] B. M. Sahoo , B. K. Banik , P. Borah , and A. Jain , “Reactive Oxygen Species (ROS): Key Components in Cancer Therapies,” Anti‐Cancer Agents in Medicinal Chemistry 22, no. 2 (2022): 215–222, 10.2174/1871520621666210608095512. 34102991

[119] A. A. Babaei , M. Rafiee , F. Khodagholi , E. Ahmadpour , and F. Amereh , “Nanoplastics‐Induced Oxidative Stress, Antioxidant Defense, and Physiological Response in Exposed Wistar Albino Rats,” Environmental Science and Pollution Research 29, no. 8 (2022): 11332–11344, 10.1007/s11356-021-15920-0. 34535860

[120] P. González‐Rodríguez , E. Zampese , K. A. Stout , et al., “Disruption of Mitochondrial Complex I Induces Progressive Parkinsonism,” Nature 599, no. 7886 (2021): 650–656, 10.1038/s41586-021-04059-0. 34732887 PMC9189968

[121] M. T. Henrich , W. H. Oertel , D. J. Surmeier , and F. F. Geibl , “Mitochondrial Dysfunction in Parkinson's Disease–A Key Disease Hallmark With Therapeutic Potential,” Molecular Neurodegeneration 18, no. 1 (2023): 83, 10.1186/s13024-023-00676-7. 37951933 PMC10640762

[122] Y. Wang , X. Zhang , W. Zhang , et al., “Melatonin Protects Dopaminergic Neurons in Paraquat‐Induced Parkinson in Rats Through PI3K/AKT/Nrf2 Pathway,” Food and Chemical Toxicology 203 (2025): 115600, 10.1016/j.fct.2025.115600. 40518038

[123] A. C. Cristóvão , D.‐H. Choi , G. Baltazar , M. F. Beal , and Y.‐S. Kim , “The Role of NADPH Oxidase 1–Derived Reactive Oxygen Species in Paraquat‐Mediated Dopaminergic Cell Death,” Antioxidants & Redox Signaling 11, no. 9 (2009): 2105–2118, 10.1089/ars.2009.2459. 19450058 PMC2935343

[124] M. S. Brandes and N. E. Gray , “NRF2 as a Therapeutic Target in Neurodegenerative Diseases,” ASN Neuro 12 (2020): 1759091419899782, 10.1177/1759091419899782. 31964153 PMC6977098

[125] Z. D. Zhang , Y. J. Yang , X. W. Liu , Z. Qin , S. H. Li , and J. Y. Li , “Aspirin Eugenol Ester Ameliorates Paraquat‐Induced Oxidative Damage Through ROS/p38‐MAPK‐Mediated Mitochondrial Apoptosis Pathway,” Toxicology 453 (2021): 152721, 10.1016/j.tox.2021.152721. 33592258

[126] M. Y. Zamanian , R. M. R. Parra , A. Soltani , et al., “Targeting Nrf2 Signaling Pathway and Oxidative Stress by Resveratrol for Parkinson's Disease: An Overview and Update on New Developments,” Molecular Biology Reports 50, no. 6 (2023): 5455–5464, 10.1007/s11033-023-08409-1. 37155008

[127] R. Jahani and H. Mohammadi , “Paraquat‐Induced Neurotoxicity: Mechanisms and Potential Therapies,” Letters in Drug Design & Discovery 22, no. 11 (2026): 100258.

[128] M. Lafuente , M. E. Rodríguez González‐Herrero , S. Romeo Villadóniga , and J. C. Domingo , “Antioxidant Activity and Neuroprotective Role of Docosahexaenoic Acid (DHA) Supplementation in Eye Diseases That Can Lead to Blindness: A Narrative Review,” Antioxidants 10, no. 3 (2021): 386, 10.3390/antiox10030386. 33807538 PMC8000043

[129] C. Gaucher , A. Boudier , J. Bonetti , I. Clarot , P. Leroy , and M. Parent , “Glutathione: Antioxidant Properties Dedicated to Nanotechnologies,” Antioxidants 7, no. 5 (2018): 62, 10.3390/antiox7050062. 29702624 PMC5981248

[130] H. M. Flafel , M. Rafatullah , J. Lalung , S. Al‐Sodies , M. A. Alshubramy , and M. A. Hussein , “Unveiling the Hazards: Comprehensive Assessment of Paraquat Herbicide's Toxicity and Health Effects,” Euro‐Mediterranean Journal for Environmental Integration 9, no. 4 (2024): 1851–1871, 10.1007/s41207-024-00537-9.

[131] B. Onur , K. Çavuşoğlu , E. Yalçin , and A. Acar , “Paraquat Toxicity in Different Cell Types of Swiss Albino Mice,” Scientific Reports 12, no. 1 (2022): 4818, 10.1038/s41598-022-08961-z. 35314741 PMC8938524

[132] R. Silva , A. F. Sobral , R. J. Dinis‐Oliveira , and D. J. Barbosa , “The Link Between Paraquat and Demyelination: A Review of Current Evidence,” Antioxidants 13, no. 11 (2024): 1354, 10.3390/antiox13111354. 39594496 PMC11590890

[133] S. da Silva , C. D. L. da Costa , A. A. Naime , D. B. Santos , M. Farina , and D. Colle , “Mechanisms Mediating the Combined Toxicity of Paraquat and Maneb in SH‐SY5Y Neuroblastoma Cells,” Chemical Research in Toxicology 37, no. 8 (2024): 1269–1282. 39058280 10.1021/acs.chemrestox.3c00389 PMC11337211

[134] S. He , Q. Ru , L. Chen , G. Xu , and Y. Wu , “Advances in Animal Models of Parkinson's Disease,” Brain Research Bulletin 215 (2024): 111024, 10.1016/j.brainresbull.2024.111024. 38969066

[135] W. Gao , J. He , L. Chen , et al., “Deciphering the Catalytic Mechanism of Superoxide Dismutase Activity of Carbon Dot Nanozyme,” Nature Communications 14, no. 1 (2023): 160, 10.1038/s41467-023-35828-2. PMC9834297 36631476

[136] O. M. Ighodaro and O. A. Akinloye , “First Line Defence Antioxidants‐Superoxide Dismutase (SOD), Catalase (CAT) and Glutathione Peroxidase (GPX): Their Fundamental Role in the Entire Antioxidant Defence Grid,” Alexandria Journal of Medicine 54, no. 4 (2018): 287–293, 10.1016/j.ajme.2017.09.001.

[137] N. Mittra , A. K. Chauhan , G. Singh , D. K. Patel , and C. Singh , “Postnatal Zinc or Paraquat Administration Increases Paraquat or Zinc‐Induced Loss of Dopaminergic Neurons: Insight Into Augmented Neurodegeneration,” Molecular and Cellular Biochemistry 467, no. 1 (2020): 27–43, 10.1007/s11010-020-03694-x. 32060784

[138] L. P. Liang , T. J. Kavanagh , and M. Patel , “Glutathione Deficiency in *Gclm* Null Mice Results in Complex I Inhibition and Dopamine Depletion Following Paraquat Administration,” Toxicological Sciences 134, no. 2 (2013): 366–373, 10.1093/toxsci/kft112. 23704229 PMC3707437

[139] P. Y. Lam and K. Ming Ko , “(−)Schisandrin B Ameliorates Paraquat‐Induced Oxidative Stress by Suppressing Glutathione Depletion and Enhancing Glutathione Recovery in Differentiated PC12 Cells,” Biofactors 37, no. 1 (2011): 51–57, 10.1002/biof.136. 21328628

[140] D. Lou , X. Chang , W. Li , Q. Zhao , Y. Wang , and Z. Zhou , “Paraquat Affects the Homeostasis of Dopaminergic System in PC12 Cells,” Pesticide Biochemistry and Physiology 103, no. 2 (2012): 81–86, 10.1016/j.pestbp.2012.04.001.

[141] M. M. Djukic , M. D. Jovanovic , M. Ninkovic , I. Stevanovic , K. Ilic , M. Curcic , and J. Vekic , “Protective Role of Glutathione Reductase in Paraquat Induced Neurotoxicity,” Chemico‐Biological Interactions 199, no. 2 (2012): 74–86, 10.1016/j.cbi.2012.05.008. 22721943

[142] S. P. Tang , S. Kuttulebbai Nainamohamed Salam , H. Jaafar , S. H. Gan , M. Muzaimi , and S. A. Sulaiman , “Tualang Honey Protects the Rat Midbrain and Lung Against Repeated Paraquat Exposure,” Oxidative Medicine and Cellular Longevity 2017, no. 1 (2017): 4605782, 10.1155/2017/4605782. 28127418 PMC5239975

[143] A. Popa‐Wagner , S. Mitran , S. Sivanesan , E. Chang , and A. M. Buga , “ROS and Brain Diseases: The Good, the Bad, and the Ugly,” Oxidative Medicine and Cellular longevity 2013, no. 1 (2013): 963520. 24381719 10.1155/2013/963520 PMC3871919

[144] M. Bandookwala and P. Sengupta , “3‐Nitrotyrosine: A Versatile Oxidative Stress Biomarker for Major Neurodegenerative Diseases,” International Journal of Neuroscience 130, no. 10 (2020): 1047–1062, 10.1080/00207454.2020.1713776. 31914343

[145] Q. Li , H. Xiao , Y. Shao , X. Chang , Y. Zhang , and Z. Zhou , “Paraquat Increases Interleukin‐1*β* in Hippocampal Dentate Gyrus to Impair Hippocampal Neurogenesis in Adult Mice,” Ecotoxicology and Environmental Safety 200 (2020): 110733, 10.1016/j.ecoenv.2020.110733. 32450442

[146] A. A. Anand , M. Khan , M. V , and D. Kar , “The Molecular Basis of Wnt/*β*‐Catenin Signaling Pathways in Neurodegenerative Diseases,” International Journal of Cell Biology 2023, no. 1 (2023): 9296092. 37780577 10.1155/2023/9296092 PMC10539095

[147] K. Li , X. Cheng , J. Jiang , et al., “The Toxic Influence of Paraquat on Hippocampal Neurogenesis in Adult Mice,” Food and Chemical Toxicology 106 (2017): 356–366, 10.1016/j.fct.2017.05.067. 28576469

[148] P. Moyano , J. Sanjuan , J. M. García , et al., “Primary Hippocampal Estrogenic Dysfunction Induces Synaptic Proteins Alteration and Neuronal Cell Death After Single and Repeated Paraquat Exposure,” Food and Chemical Toxicology 136 (2020): 110961, 10.1016/j.fct.2019.110961. 31715309

[149] Z. Yang , Y. Shao , Y. Zhao , et al., “Endoplasmic Reticulum Stress‐Related Neuroinflammation and Neural Stem Cells Decrease in Mice Exposure to Paraquat,” Scientific Reports 10, no. 1 (2020): 17757, 10.1038/s41598-020-74916-x. 33082501 PMC7576831

[150] S. J. Chinta , G. Woods , M. Demaria , et al., “Cellular Senescence is Induced by the Environmental Neurotoxin Paraquat and Contributes to Neuropathology Linked to Parkinson's Disease,” Cell Reports 22, no. 4 (2018): 930–940, 10.1016/j.celrep.2017.12.092. 29386135 PMC5806534

[151] R. Reis E Silva , T. M. Portal , N. D. S. Nogueira , T. D. S. Nogueira , A. A. Mello , and C. Monteiro‐de‐Barros , “Paraquat Neurotoxicity: Oxidative Stress and Neuronal Dysfunction in the Ascidian Brain,” Comparative Biochemistry and Physiology Part C: Toxicology & Pharmacology 290 (2025): 110128. 39855440 10.1016/j.cbpc.2025.110128

[152] K. Wang , C. Zhang , B. Zhang , G. Li , G. Shi , Q. Cai , and M. Huang , “Gut Dysfunction May be the Source of Pathological Aggregation of Alpha‐Synuclein in the Central Nervous System Through Paraquat Exposure in Mice,” Ecotoxicology and Environmental Safety 246 (2022): 114152, 10.1016/j.ecoenv.2022.114152. 36201918

[153] A. Masato , N. Plotegher , D. Boassa , and L. Bubacco , “Impaired Dopamine Metabolism in Parkinson's Disease Pathogenesis,” Molecular Neurodegeneration 14, no. 1 (2019): 35, 10.1186/s13024-019-0332-6. 31488222 PMC6728988

[154] P. M. Rappold , M. Cui , A. S. Chesser , et al., “Paraquat Neurotoxicity is Mediated by the Dopamine Transporter and Organic Cation Transporter‐3,” Proceedings of the National Academy of Sciences 108, no. 51 (2011): 20766–20771, 10.1073/pnas.1115141108. PMC3251116 22143804

[155] S. Delcambre , Y. Nonnenmacher , and K. Hiller , “Dopamine Metabolism and Reactive Oxygen Species Production,” in Mitochondrial Mechanisms of Degeneration and Repair in Parkinson's Disease (Springer International Publishing, 2016), 25–47, 10.1007/978-3-319-42139-110.1007/978-3-319-42139-1_2.

[156] S. Isik , B. Yeman Kiyak , R. Akbayir , R. Seyhali , and T. Arpaci , “Microglia Mediated Neuroinflammation in Parkinson's Disease,” Cells 12, no. 7 (2023): 1012, 10.3390/cells12071012. 37048085 PMC10093562

[157] D. Kempuraj , R. Thangavel , P. A. Natteru , G. P. Selvakumar , D. Saeed , H. Zahoor , and A. Zaheer , “Neuroinflammation Induces Neurodegeneration,” Journal of Neurology, Neurosurgery and Spine 1, no. 1 (2016): 1003. 28127589 PMC5260818

[158] S. A. Adefegha , O. B. Ogunsuyi , O. P. Aro , and G. Oboh , “Effect of Paraquat‐Induced Toxicity and the Therapeutic Potentials of *Moringa oleifera* and *Moringa stenopetala* Leaf Alkaloid Extracts,” Pharmacological Research—Reports 4 (2025): 100049, 10.1016/j.prerep.2025.100049.

[159] H. M. Yang , Y. L. Wang , C. Y. Liu , Y. T. Zhou , and X. F. Zhang , “A Time‐Course Study of Microglial Activation and Dopaminergic Neuron Loss in the Substantia Nigra of Mice With Paraquat‐Induced Parkinson's Disease,” Food and Chemical Toxicology 164 (2022): 113018, 10.1016/j.fct.2022.113018. 35430334

[160] Y. Sun , J. Zheng , Y. Xu , and X. Zhang , “Paraquat‐Induced Inflammatory Response of Microglia Through HSP60/TLR4 Signaling,” Human & Experimental Toxicology 37, no. 11 (2018): 1161–1168, 10.1177/0960327118758152. 29441825

[161] T. Tong , W. Duan , Y. Xu , et al., “Paraquat Exposure Induces Parkinsonism by Altering Lipid Profile and Evoking Neuroinflammation in the Midbrain,” Environment International 169 (2022): 107512, 10.1016/j.envint.2022.107512. 36108500

[162] S. Barua , J. Y. Kim , M. A. Yenari , and J. E. Lee , “The Role of NOX Inhibitors in Neurodegenerative Diseases,” IBRO Reports 7 (2019): 59–69, 10.1016/j.ibror.2019.07.1721. 31463415 PMC6709343

[163] D. Furutama , S. Matsuda , Y. Yamawaki , et al., “IL‐6 Induced by Periodontal Inflammation Causes Neuroinflammation and Disrupts the Blood–Brain Barrier,” Brain Sciences 10, no. 10 (2020): 679, 10.3390/brainsci10100679. 32992470 PMC7599694

[164] J. S. von Tobel , D. Zoia , J. Althaus , P. Antinori , J. Mermoud , H. S. Pak , and F. Monnet‐Tschudi , “Immediate and Delayed Effects of Subchronic Paraquat Exposure During an Early Differentiation Stage in 3D‐Rat Brain Cell Cultures,” Toxicology Letters 230, no. 2 (2014): 188–197. 24521700 10.1016/j.toxlet.2014.02.001

[165] M. P. Singh , Twinkle , and R. Himalian , “Generation of Neurodegenerative Phenotype Using *Drosophila melanogaster* Through Paraquat Treatment and an Amelioration by *Tinospora cordifolia* (Giloy),” Research Journal of Pharmacy and Technology 14, no. 6 (2021): 3056–3062, 10.52711/0974-360X.2021.00534.

[166] C. Torres‐Rojas , D. Zhuang , P. Jimenez‐Carrion , et al., “Systems Genetics and Systems Biology Analysis of Paraquat Neurotoxicity in BXD Recombinant Inbred Mice,” Toxicological Sciences 176, no. 1 (2020): 137–146, 10.1093/toxsci/kfaa050. 32294219 PMC7357179

[167] U. Maitra , M. N. Scaglione , S. Chtarbanova , and J. M. O'Donnell , “Innate Immune Responses to Paraquat Exposure in a Drosophila Model of Parkinson's Disease,” Scientific Reports 9, no. 1 (2019): 12714, 10.1038/s41598-019-48977-6. 31481676 PMC6722124

[168] M. Singh , V. Murthy , and C. Ramassamy , “Neuroprotective Mechanisms of the Standardized Extract of *Bacopa monniera* in a Paraquat/Diquat‐Mediated Acute Toxicity,” Neurochemistry International 62, no. 5 (2013): 530–539, 10.1016/j.neuint.2013.01.030. 23402822

[169] E. N. Mangano , D. Litteljohn , R. So , et al., “Interferon‐γ Plays a Role in Paraquat‐Induced Neurodegeneration Involving Oxidative and Proinflammatory Pathways,” Neurobiology of Aging 33, no. 7 (2012): 1411–1426, 10.1016/j.neurobiolaging.2011.02.016. 21482445

[170] C. Rudyk , Z. Dwyer , and S. Hayley , CLINT membership , “Leucine‐Rich Repeat Kinase‐2 (LRRK2) Modulates Paraquat‐Induced Inflammatory Sickness and Stress Phenotype,” Journal of Neuroinflammation 16, no. 1 (2019): 120, 10.1186/s12974-019-1483-7. 31174552 PMC6554960

[171] C. Rudyk , Z. Dwyer , J. McNeill , N. Salmaso , K. Farmer , N. Prowse , and S. Hayley , “Chronic Unpredictable Stress Influenced the Behavioral but not the Neurodegenerative Impact of Paraquat,” Neurobiology of Stress 11 (2019): 100179, 10.1016/j.ynstr.2019.100179. 31304199 PMC6599913

[172] M. Huang , Q. Cai , Y. Xu , et al., “Paraquat Affects the Differentiation of Neural Stem Cells and Impairs the Function of Vascular Endothelial Cells: A Study of Molecular Mechanism,” Environmental Toxicology 34, no. 4 (2019): 548–555, 10.1002/tox.22723. 30698896

[173] M. Huang , Y. Li , K. Wu , S. Hao , Q. Cai , Z. J. Zhou , and H. F. Yang , “Effects of Environmental Chemicals on the Proliferation and Differentiation of Neural Stem Cells,” Environmental Toxicology 34, no. 12 (2019): 1285–1291, 10.1002/tox.22829. 31400064

[174] N. A. El‐Boghdady , N. F. Abdeltawab , and M. M. Nooh , “Resveratrol and Montelukast Alleviate Paraquat‐Induced Hepatic Injury in Mice: Modulation of Oxidative Stress, Inflammation, and Apoptosis,” Oxidative Medicine and Cellular Longevity 2017, no. 1 (2017): 9396425, 10.1155/2017/9396425. 29201275 PMC5671749

[175] B. Dilberger , S. Baumanns , F. Schmitt , T. Schmiedl , M. Hardt , U. Wenzel , and G. P. Eckert , “Mitochondrial Oxidative Stress Impairs Energy Metabolism and Reduces Stress Resistance and Longevity of *C. elegans* ,” Oxidative Medicine and Cellular Longevity 2019, no. 1 (2019): 6840540. 31827694 10.1155/2019/6840540 PMC6885289

[176] N. S. Erekat , Apoptosis and Its Role in Parkinson's Disease (Exon Publications, 2018), 65–82.

[177] Q. Fei , A. L. McCormack , D. A. Di Monte , and D. W. Ethell , “Paraquat Neurotoxicity is Mediated by a Bak‐Dependent Mechanism,” Journal of Biological Chemistry 283, no. 6 (2008): 3357–3364, 10.1074/jbc.M708451200. 18056701

[178] X. Liu , Q. Wu , J. Wu , et al., “Microglia‐Derived Exosomal circZNRF1 Alleviates Paraquat‐Induced Neuronal Cell Damage via miR‐17‐5p,” Ecotoxicology and Environmental Safety 263 (2023): 115356, 10.1016/j.ecoenv.2023.115356. 37591128

[179] L. Shan , H. J. Heusinkveld , K. C. Paul , S. Hughes , S. K. Darweesh , B. R. Bloem , and J. R. Homberg , “Towards Improved Screening of Toxins for Parkinson's Risk,” npj Parkinson's Disease 9, no. 1 (2023): 169, 10.1038/s41531-023-00615-9. PMC10730534 38114496

[180] D.‐T. Ju , K. Sivalingam , W.‐W. Kuo , et al., “Effect of Vasicinone Against Paraquat‐Induced MAPK/p53‐Mediated Apoptosis via the IGF‐1R/PI3K/AKT Pathway in a Parkinson's Disease‐Associated SH‐SY5Y Cell Model,” Nutrients 11, no. 7 (2019): 1655, 10.3390/nu11071655. 31331066 PMC6682869

[181] A. Ray Chaudhuri and A. Nussenzweig , “The Multifaceted Roles of PARP1 in DNA Repair and Chromatin Remodelling,” Nature Reviews Molecular Cell Biology 18, no. 10 (2017): 610–621. 28676700 10.1038/nrm.2017.53 PMC6591728

[182] J. Han , Z. Zhang , S. Yang , J. Wang , X. Yang , and D. Tan , “Betanin Attenuates Paraquat‐Induced Liver Toxicity Through a Mitochondrial Pathway,” Food and Chemical Toxicology 70 (2014): 100–106, 10.1016/j.fct.2014.04.038. 24799198

[183] B. Aryal and Y. Lee , “Disease Model Organism for Parkinson Disease: *Drosophila melanogaster* ,” BMB Reports 52, no. 4 (2019): 250–258, 10.5483/BMBRep.2019.52.4.204. 30545438 PMC6507844

[184] H. Yang , Y. Yang , L. Yang , Z. Li , L. Lyu , and X. Zhang , “Activated Microglia Contribute to Paraquat Neurotoxicity Through Neuroinflammation and Regulation of Phenotypic Polarization of Astrocytes,” Ecotoxicology and Environmental Safety 316 (2026): 120120, 10.1016/j.ecoenv.2026.120120. 41966020

[185] S. Gollamudi , A. Johri , N. Y. Calingasan , L. Yang , O. Elemento , and M. F. Beal , “Concordant Signaling Pathways Produced by Pesticide Exposure in Mice Correspond to Pathways Identified in Human Parkinson's Disease,” PLoS ONE 7, no. 5 (2012): e36191, 10.1371/journal.pone.0036191. 22563483 PMC3341364

[186] J. Kakish , D. Lee , and J. S. Lee , “Drugs That Bind to *α*‐Synuclein: Neuroprotective or Neurotoxic?,” ACS Chemical Neuroscience 6, no. 12 (2015): 1930–1940, 10.1021/acschemneuro.5b00172. 26378986

[187] Y. Weng , Y. Zhang , Y. Li , et al., “Single‐Cell RNA‐Sequencing of Cellular Heterogeneity and Pathogenic Mechanisms in Paraquat‐Induced Parkinson's Disease With Depression,” Ecotoxicology and Environmental Safety 273 (2024): 116169, 10.1016/j.ecoenv.2024.116169. 38447518

[188] C. Zhang , G. Shi , Q. Meng , et al., “An Approach Based on a Combination of Toxicological Experiments and In Silico Predictions to Investigate the Adverse Outcome Pathway (AOP) of Paraquat Neuro‐Immunotoxicity,” Journal of Hazardous Materials 473 (2024): 134607, 10.1016/j.jhazmat.2024.134607. 38761765

[189] Y. Zhang , Y. Jiang , Y. Li , et al., “Brain Single‐Cell Transcriptomics Highlights Comorbidity‐Related Cell Type‐Specific Changes of Parkinson's Disease With Major Depressive Disorder After Paraquat Exposure,” Ecotoxicology and Environmental Safety 286 (2024): 117193, 10.1016/j.ecoenv.2024.117193. 39413649

[190] Y. Yang , Y. Xu , Q. Meng , X. Wang , Y. Tian , L. Lyu , and X. Zhang , “Paraquat Induces Neuroinflammation Through Glycolytic Reprogramming in Microglia: Evidence From Network Toxicology Analysis and In Vitro Experiments,” Chemico‐Biological Interactions 432 (2026): 112037, 10.1016/j.cbi.2026.112037. 41839307

[191] H. A. Craddock , D. Huang , P. C. Turner , L. Quirós‐Alcalá , and D. C. Payne‐Sturges , “Trends in Neonicotinoid Pesticide Residues in Food and Water in the United States, 1999–2015,” Environmental Health 18, no. 1 (2019): 7, 10.1186/s12940-018-0441-7. 30634980 PMC6330495

[192] C. Coughlan , D. I. Walker , K. M. Lohr , J. R. Richardson , L. M. Saba , W. M. Caudle , and J. R. Roede , “Comparative Proteomic Analysis of Carbonylated Proteins From the Striatum and Cortex of Pesticide‐Treated Mice,” Parkinson's Disease 2015, no. 1 (2015): 812532. 10.1155/2015/812532 PMC4546751 26345149

[193] J. Wills , J. Credle , A. W. Oaks , V. Duka , J. H. Lee , J. Jones , and A. Sidhu , “Paraquat, But Not Maneb, Induces Synucleinopathy and Tauopathy in Striata of Mice Through Inhibition of Proteasomal and Autophagic Pathways,” PLoS ONE 7, no. 1 (2012): e30745, 10.1371/journal.pone.0030745. 22292029 PMC3264632

[194] J. Ma , Y. Li , W. Li , and X. Li , “Hepatotoxicity of Paraquat on Common Carp (*Cyprinus carpio* L.),” Science of the Total Environment 616 (2018): 889–898, 10.1016/j.scitotenv.2017.10.231. 29107372

[195] H. Liu , C. Koros , T. Strohäker , et al., “A Novel SNCA A30G Mutation Causes Familial Parkinsonʼs Disease,” Movement Disorders 36, no. 7 (2021): 1624–1633, 10.1002/mds.28534. 33617693

[196] A. Kumar , F. Leinisch , M. B. Kadiiska , J. Corbett , and R. P. Mason , “Formation and Implications of Alpha‐Synuclein Radical in Maneb‐and Paraquat‐Induced Models of Parkinson's Disease,” Molecular neurobiology 53, no. 5 (2016): 2983–2994, 10.1007/s12035-015-9179-1. 25952542 PMC4637265

